# Palmitoylation landscapes across human cancers reveal a role of palmitoylation in tumorigenesis

**DOI:** 10.1186/s12967-023-04611-8

**Published:** 2023-11-17

**Authors:** Yue Kong, Yugeng Liu, Xianzhe Li, Menglan Rao, Dawei Li, Xiaolan Ruan, Shanglin Li, Zhenyou Jiang, Qiang Zhang

**Affiliations:** 1 Department of Microbiology and Immunology, Basic Medicine College, Jinan University, No.601, West Huangpu Avenue, Guangzhou, 510632 Guangdong China; 2 Key Laboratory of Ministry of Education for Viral Pathogenesis and Infection Prevention and Control, Jinan University, Guangzhou, 510632 China; 3 Shenzhen Branch, Guangdong Laboratory of Lingnan Modern Agriculture, Genome Analysis Laboratory of the Ministry of Agriculture, Agricultural Genomics Institute at Shenzhen, Chinese Academy of Agricultural Sciences, Shenzhen, 518124 China; 4 Division of Clinical Epidemiology and Aging Research, German Cancer Research Center (DKFZ), 69120 Heidelberg, Germany; 5 Guangdong Provincial Key Laboratory of Virology, Institute of Medical Microbiology, Jinan University, Guangzhou, 510632 China; 6 Zhumadian Central Hospital, Huanghuai University, Zhumadian, 463000 China; 7 Molecular Cancer Research Center, School of Medicine, Shenzhen Campus of Sun Yat-sen University, Sun Yat-sen University, No.66, Gongchang Road, Guangming District, Shenzhen, 518107 Guangdong China

**Keywords:** Palmitoylation, Cancer, DNA methylation, c-Myc, Immunotherapy, Small molecular

## Abstract

**Background:**

Protein palmitoylation, which is catalyzed by palmitoyl-transferase and de-palmitoyl-transferase, plays a crucial role in various biological processes. However, the landscape and dynamics of protein palmitoylation in human cancers are not well understood.

**Methods:**

We utilized 23 palmitoyl-acyltransferases and seven de-palmitoyl-acyltransferases as palmitoylation-related genes for protein palmitoylation analysis. Multiple publicly available datasets were employed to conduct pan-cancer analysis, examining the transcriptome, genomic alterations, clinical outcomes, and correlation with c-Myc (Myc) for palmitoylation-related genes. Real-time quantitative PCR and immunoblotting were performed to assess the expression of palmitoylation-related genes and global protein palmitoylation levels in cancer cells treated with Myc depletion or small molecule inhibitors. Protein docking and drug sensitivity analyses were employed to predict small molecules that target palmitoylation-related genes.

**Results:**

We identified associations between palmitoylation and cancer subtype, stage, and patient survival. We discovered that abnormal DNA methylation and oncogenic Myc-driven transcriptional regulation synergistically contribute to the dysregulation of palmitoylation-related genes. This dysregulation of palmitoylation was closely correlated with immune infiltration in the tumor microenvironment and the response to immunotherapy. Importantly, dysregulated palmitoylation was found to modulate canonical cancer-related pathways, thus influencing tumorigenesis. To support our findings, we performed a proof-of-concept experiment showing that depletion of Myc led to reduced expression of most palmitoylation-related genes, resulting in decreased global protein palmitoylation levels. Through mass spectrometry and enrichment analyses, we also identified palmitoyl-acyltransferases ZDHHC7 and ZDHHC23 as significant contributors to mTOR signaling, DNA repair, and immune pathways, highlighting their potential roles in tumorigenesis. Additionally, our study explored the potential of three small molecular (BI-2531, etoposide, and piperlongumine) to modulate palmitoylation by targeting the expression or activity of palmitoylation-related genes or enzymes.

**Conclusions:**

Overall, our findings underscore the critical role of dysregulated palmitoylation in tumorigenesis and the response to immunotherapy, mediated through classical cancer-related pathways and immune cell infiltration. Additionally, we propose that the aforementioned three small molecule hold promise as potential therapeutics for modulating palmitoylation, thereby offering novel avenues for cancer therapy.

**Supplementary Information:**

The online version contains supplementary material available at 10.1186/s12967-023-04611-8.

## Materials and methods

### Cell culture and transfection

Human HCT116, SW48, HT29 and HEK293T cells were derived from the American Type Culture Collection (ATCC). HEK293T cells were cultured in DMEM medium (Gibco, NY, USA) supplemented with 10% fetal bovine serum (Gibco, NY, USA) and 1% penicillin–streptomycin (Gibco, CA, USA) at 37 °C in a 5% CO_2_ incubator. For transfection, after growing to 70% confluence, cells were transfected using Lipofectamine 3000 (Invitrogen, Carlsbad, CA) according to the manufacturer's instructions when they reached 70% confluence.

### Reagents and plasmids

Anti-Flag agarose beads (23101) were purchased from Selleck (Houston, USA). Anti-HA agarose beads (KTSM1305) were obtained from Shenzhen KangTi Life Technology (Shenzhen, China). The human ZDHHC7 and ZDHHC23 plasmids were generously provided by Dr. Guojun Zhao from The First Affiliated Hospital of Zhengzhou University, China. Human Myc shRNAs were purchased from Miaoling Biology (Whuhan, China). The shRNA sequences used in the study can be found in Additional file 1: Table S1. Small molecules (BI2536 (T6173), etoposide (T0132), piperlongumine (T6947)) were purchased from Topscience Biotech (Shanghai, China). Hydroxylamine (HAM, 467804), N-Ethylmaleimide (NEM, A600450-0005) and BMCC-biotin ((1-Biotinamido)-4-[4'-(mal-eimidomethyl)cyclohexanecarboxamido]hexane, C100222-0050) were purchased from Sangon Biotech (Shanghai, China).

### Data and resources

RNA-Seq, somatic mutation, DNA methylation, copy number variant (CNV), and clinical data were from The Cancer Genome Atlas (TCGA) or Genotype-Tissue Expression (GTEx). Immunogenomic analysis was performed by the Immune Cell Abundance Identifier (ImmuCellAI) [1, 2] algorithm with 24 immune cells. Information on small molecule drugs is from the Cancer Therapeutics Response Portal (CTRP) [3] and Genomics of Drug Sensitivity in Cancer (GDSC) website. These data were integrated into the Gene Set Cancer Analysis (GSCA) for pan-cancer analysis in this study [4]. All TCGA cancer types included in the study are listed in Additional file 1: Table S2.

### Gene Set Variation Analysis (GSVA)

GSVA analysis was performed on nine cancer types (HNSC, LUSC, COAD, STAD, LUAD, GBM, BRCA, KIRC, BLCA) with available subtype information. The analysis was conducted using the R package GSVA, following previously described methods [4]. In this analysis, the GSVA score, which represents the variation of a gene set activity in a specific cancer sample population, was calculated. The GSVA score reflects the integrated level of gene set expression and is positively correlated with the expression of the gene set. By comparing the GSVA scores of tumor tissues with those of adjacent samples, we were able to determine if the overall expression of a particular gene set was higher in the tumor tissues.

### Differential expression analysis

Differential expression analysis was conducted for 14 TCGA cancer types (THCA, KIRP, BLCA, LIHC, HNSC, BRCA, LUAD, PRAD, ESCA, KICH, LUSC, KIRC, STAD, COAD) with more than 10 paired tumor and normal samples. This analysis was based on normalized and batch corrected RSEM mRNA expression data. The fold change was calculated by taking the mean expression in tumor samples divided by the mean expression in normal samples. The statistical significance was determined using a t-test, and the resulting *P* values were adjusted for multiple testing using the false discovery rate (FDR) method.

### Differential methylation analysis

Differential methylation analysis was performed using Illumina Human Methylation 450k level data obtained from the TCGA database. This analysis was conducted for 14 different types of cancer, including THCA, KIRP, BLCA, LIHC, HNSC, BRCA, LUAD, PRAD, ESCA, KICH, LUSC, KIRC, STAD, and COAD. Only cancer types with more than 10 paired tumor and normal samples were included in the analysis. Since a region of a gene can have multiple methylation sites, there are multiple tags that store methylation levels at each site. Before conducting the differential methylation analysis, we performed correlation analysis to filter out the sites that were most negatively correlated with gene expression. This was done to ensure that only relevant sites were included in the analysis. To calculate the differential methylation, we used the ratio of the mean methylation levels in tumor samples to the mean methylation levels in normal samples. The statistical significance was determined using a t-test, and the P values were further adjusted using the FDR method.

### CNV analysis

CNV analysis was performed using data from 11,495 samples obtained from the TCGA database. The data was processed using GISTIC2.0, a program that aims to identify regions of amplification or deletion that are significantly altered in patients. The CNV data is represented by different values: -2 or Deep Deletion indicates a deep loss, possibly a homozygous deletion; -1 or Shallow Deletion indicates a shallow loss, possibly a heterozygous deletion; 0 represents diploid status; 1 or Gain indicates a low-level gain, possibly a heterozygous amplification; 2 or Amplification indicates a high-level amplification, possibly a homozygous amplification.

### Mutation analysis

To analyze the mutations, we obtained mutation data from the TCGA database consisting of 10,234 samples. We focused on seven types of mutations, namely Missense_Mutation, Nonsense_Mutation, Frame_Shift_Ins, Splice_Site, Frame_Shift_Del, In_Frame_Del, and In_Frame_Ins, which are considered deleterious mutations. Additionally, we included non-deleterious mutations such as Silent, Intron, IGR, 3'UTR, 5'UTR, 3'Flank, and 5'Flank in our analysis.

### Survival analysis

Survival analysis was conducted using clinical data from 33 cancer types from TCGA tumor samples. The survival types analyzed included overall survival (OS), progression-free survival (PFS), disease-specific survival (DSS), and disease-free interval (DFI) [5]. Some uncensored data was excluded, and samples of tumors at risk of competing cancer deaths were filtered out. Regarding data availability, OS data is available for all cancer types, while PFS data is not available for LAML. DSS data is also not available for LAML. and the DFI data are not available for LAML, SKCM, THYM, and UVM. Additionally, samples of tumors at risk of competing cancer deaths were excluded from the analysis.

For expression/methylation and survival analysis, mRNA/methylation data were combined with clinical survival data based on sample barcode. Tumor samples were divided into a high expression/methylation group and a low expression/methylation group based on the median mRNA/methylation value. Cox Proportional-Hazards models and log-rank tests were performed for each gene or gene set in each cancer type.

For mutation and survival analysis, mutation data were combined with clinical survival data based on sample barcode. Tumor samples were classified as the Mutant group when deleterious mutations occurred in specific genes. Cox Proportional-Hazards models and log-rank tests were performed to compare the survival difference between the Wild-Type (WT) and Mutant groups.

### Immune infiltration analysis

To assess immune infiltration and mRNA expression analysis, the Spearman correlation was employed to determine the relationship between gene mRNA expression or gene sets and the infiltration of immune cells. The abundance of 24 immune cell types was estimated using gene set signatures, and the infiltrates of these immune cells were evaluated through ImmuCellAI.

### Real-time quantitative PCR (RT-qPCR)

The RT-qPCR assays were performed according to the methods described in a previous study [6]. Total RNA was extracted from cells and subjected to reverse transcription. Then, qPCR was conducted using the SYBR Green Supermix (Bio-Rad, Hercules, CA) and standard protocols. The primer sequences used in the study can be found in Additional file 1: Table S1. GAPDH was utilized as the internal control for normalization purposes.

### Global palmitoylation detection

To detect global protein palmitoylation, we employed the acyl-biotin exchange (ABE) method [7]. Briefly, cells were lysed using a lysis buffer, and 80 μL of the resulting supernatant was saved as the input sample. The remaining supernatant was utilized for the ABE experiments. The detection of global protein palmitoylation was performed using HRP-conjugated streptavidin (Sangon Biotech, diluted 1:200 in 0.5% BSA). All antibodies used in the study are listed in Additional file 1: Table S3.

### Pathway activity analysis

To analyze pathway activity, we utilized RPPA data from the TCPA database. We calculated pathway activity scores for 10 cancer-associated pathways including TSC/mTOR, RTK, RAS/MAPK, PI3K/AKT, Hormone ER, Hormone AR, EMT, DNA Damage Response, Cell Cycle, and Apoptosis pathways. These scores were calculated using 7876 samples from the TCGA database across 32 different cancer types. The RPPA data from RBN were first centered on the median and then normalized by the standard deviation for each component in all samples to obtain relative protein levels. The pathway score for each sample is calculated as the sum of the relative protein levels of all positive regulatory components minus the relative protein levels of negative regulatory components within a given pathway [8].

To analyze gene set and pathway activity, we initially performed GSVA analysis on the gene set. GSVA scores were calculated using the R package GSVA and represent the variation of gene set activity in a specific population of cancer samples in an unsupervised manner. The GSVA score reflects the overall expression level of the gene set and is positively correlated with gene set expression.

For gene expression and pathway activity analysis, we categorized the samples into high and low groups based on the median gene expression level. The differences in pathway activity score (PAS) between these two groups were determined using a student t-test, and the resulting *P* values were adjusted using the FDR method. A significance level of FDR ≤0.05 was considered statistically significant. If the PAS (Gene A High expression) > PAS (Gene A Low expression), we inferred that Gene A may have an activating effect on the pathway. Conversely, if the PAS (Gene A High expression) < PAS (Gene A Low expression), Gene A may have an inhibitory effect on the pathway [9]. The global percentage of cancers in which a gene has an effect on the pathway among 32 cancer types is calculated as the percentage (number of cancer types in which the gene is activated or inhibited/32 * 100%).

### Immunoprecipitation and mass spectrometry analysis

Immunoprecipitation and mass spectrometry analysis were conducted following previously described methods [6]. HEK293T cells transfected with the ZDHHC23/7 plasmid were lysed in 1 mL of lysis buffer. Anti-Flag/HA agarose beads were washed with 1 mL of lysis buffer three times, and then 0.95 mL of cell lysate was added to the respective groups and incubated overnight at 4 °C. The following day, the agarose beads were centrifuged and washed three times with a wash buffer. The beads were then mixed with a 2 × SDS sample buffer. Lysate samples were boiled for 10 min, followed by SDS-PAGE and Coomassie Blue staining. The bands were excised, subjected to in-gel trypsin digestion, and dried for mass spectrometry analysis, as previously described [10].

### CTRP and GDSC drug sensitivity analysis

To analyze drug sensitivity, we obtained the IC50 values of 481 small molecules in 1001 cell lines from the CTRP database. We also downloaded the corresponding mRNA gene expression data. To assess the correlation between mRNA expression and drug sensitivity, we performed Pearson correlation analysis by combining the mRNA expression and drug sensitivity data. The resulting *P* values were adjusted for FDR. To validate our findings, we conducted additional drug sensitivity analysis using the GDSC database. The results were presented using the Assistant for Clinical Bioinformatics website (https://www.aclbi.com/static/index.html#/drug_allergy).

### Protein structure acquisition

The crystal structures of LYPLA1 (PDB ID: 5SYM), LYPLA2 (PDB ID: 5SYN), PPT1 (PDB ID: 3GRO), and PPT2 (PDB ID: 1PJA) were obtained from the Protein Data Bank (PDB). For proteins without available crystal structures, we utilized the AlphaFold2 tool [11] to predict their structures. Additionally, the structures of three small molecules (BI2536, etoposide, piperlongumine) were retrieved from PubChem and underwent molecular energy minimization using chem3D 20.0 software. The Vina force field was applied to the proteins, while the small molecules were optimized with polar hydrogen atoms using the Autodock tool.

### Determination of protein active pockets and protein–ligand docking

To determine the active pockets in the proteins, we used CavityPlus 2022 [12]. The identified active pockets were then refined based on the enzymatic regions of the proteins. Protein–ligand docking studies were conducted using Autodock Vina 2021 [13], which allowed us to calculate the binding energy between the protein and small molecules. The resulting conformations from the docking were visualized using Pymol, enabling us to analyze the intermolecular interactions involved in the protein–ligand interactions.

### Statistical analysis

The *P* value was calculated using the t-test and adjusted for multiple comparisons using the FDR method. Details of the specific statistical methods used can be found in the corresponding methods section or figure legends. For general analysis and visualization, we utilized the GSCA [4]. A significance level of FDR ≤ 0.05 or P < 0.05 was considered statistically significant.

## Introduction

Protein palmitoylation, a reversible post-translational modification, plays a crucial role in altering the localization, stability, and function of proteins [14–18]. The zinc finger DHHC-type containing (ZDHHC) family, consisting of 23 distinct proteins in mammals, catalyzes this reversible modification [14, 15]. On the other hand, de-palmitoylation is typically mediated by three distinct families that include seven genes [16–18]. Previous research from our group has demonstrated the promotion of colorectal cancer progression by palmitoylated β-catenin [7]. Moreover, emerging evidence suggests that palmitoyl-acyltransferases and de-palmitoyl-lacyltransferases are associated with various aspects of carcinogenesis, cancer cell growth, survival, and treatment resistance [18, 19]. Consequently, enzymes involved in modulating protein palmitoylation hold great promise as potential targets for cancer therapy. Despite this potential, the landscape of palmitoylation in human cancers has yet to be fully described.

The transcription factor c-Myc (Myc) is a well-known oncogene frequently activated in various human cancers. Numerous studies have demonstrated its role in tumor initiation and progression through the regulation of multiple biological processes [20–22]. Recently, our own research has revealed that Myc can contribute to tumorigenesis by modulating GTP metabolic reprogramming [23]. These oncogenic phenotypes of Myc are primarily attributed to its ability to activate or repress gene transcription. Given the substantial influence of Myc on gene expression, it is reasonable to question whether Myc also affects global palmitoylation profiles by regulating the transcription of genes involved in palmitoylation. Investigating whether Myc influences the expression of these palmitoylation-related genes (23 palmitoyl-acyltransferases and seven de-palmitoyl-acyltransferases) in human cancers may provide valuable insights.

Tumor-infiltrating immune cells play a vital role in the tumor microenvironment and have been implicated in tumor progression and response to immunotherapy [24]. Numerous studies have demonstrated that the extent of immune cell infiltration within tumors is associated with clinical outcomes and response to immunotherapy in various cancer types [24–31]. Furthermore, recent investigations have highlighted the significance of palmitoylation in modulating CD8^+^ T cell infiltration levels in tumor tissues, with palmitoylation of PD-L1 and IFNGR1 emerging as key factors [19, 32]. These findings underscore the critical role of palmitoylation in immunotherapy. To gain a more comprehensive understanding, it is crucial to establish a detailed landscape of palmitoylation and immune cell infiltration in human cancers.

Tumorigenesis is frequently associated with the dysregulation of signaling pathways, including TSC/mTOR, RTK, RAS/MAPK, PI3K/AKT, Hormone ER, Hormone AR, EMT, DNA Damage Response, Cell Cycle, and Apoptosis pathways. These pathways play crucial roles in cancer development and progression. Recent studies have demonstrated that palmitoylation actively regulates several of these cancer-related pathways, impacting tumorigenesis [33–35]. To enhance our understanding of tumorigenesis, it is essential to establish a comprehensive landscape that delineates the involvement of palmitoylation in cancer-related pathways across different types of human cancers.

In this study, we present a comprehensive analysis of the palmitoylation landscape in human cancers. We highlight the relationship between Myc and palmitoylation and demonstrate that dysregulated palmitoylation plays a pivotal role in driving tumorigenesis by modulating classical cancer-related pathways and immune infiltration. Moreover, we identify three small molecular compounds, namely BI-2531, etoposide, and piperlongumine, that have the potential to modulate palmitoylation and could serve as promising candidates for cancer therapy.

## Results

### Dysregulated palmitoylation in human cancers

To investigate the landscape of palmitoylation in human cancers, we analyzed the expression of palmitoylation-related genes using data from the GTEx and TCGA databases, respectively (Additional file 1: Table S4). Initially, we evaluated the expression patterns of these genes across diverse human tissues using the GTEx database and found that most genes, except for a few exceptions such as ZDHHC18, ZDHHC19, and PPT2, exhibited relatively consistent expression levels (Additional file 2: Fig. S1). This suggests that protein palmitoylation may play a comparable role in multiple human tissues.

**Fig. 1:**
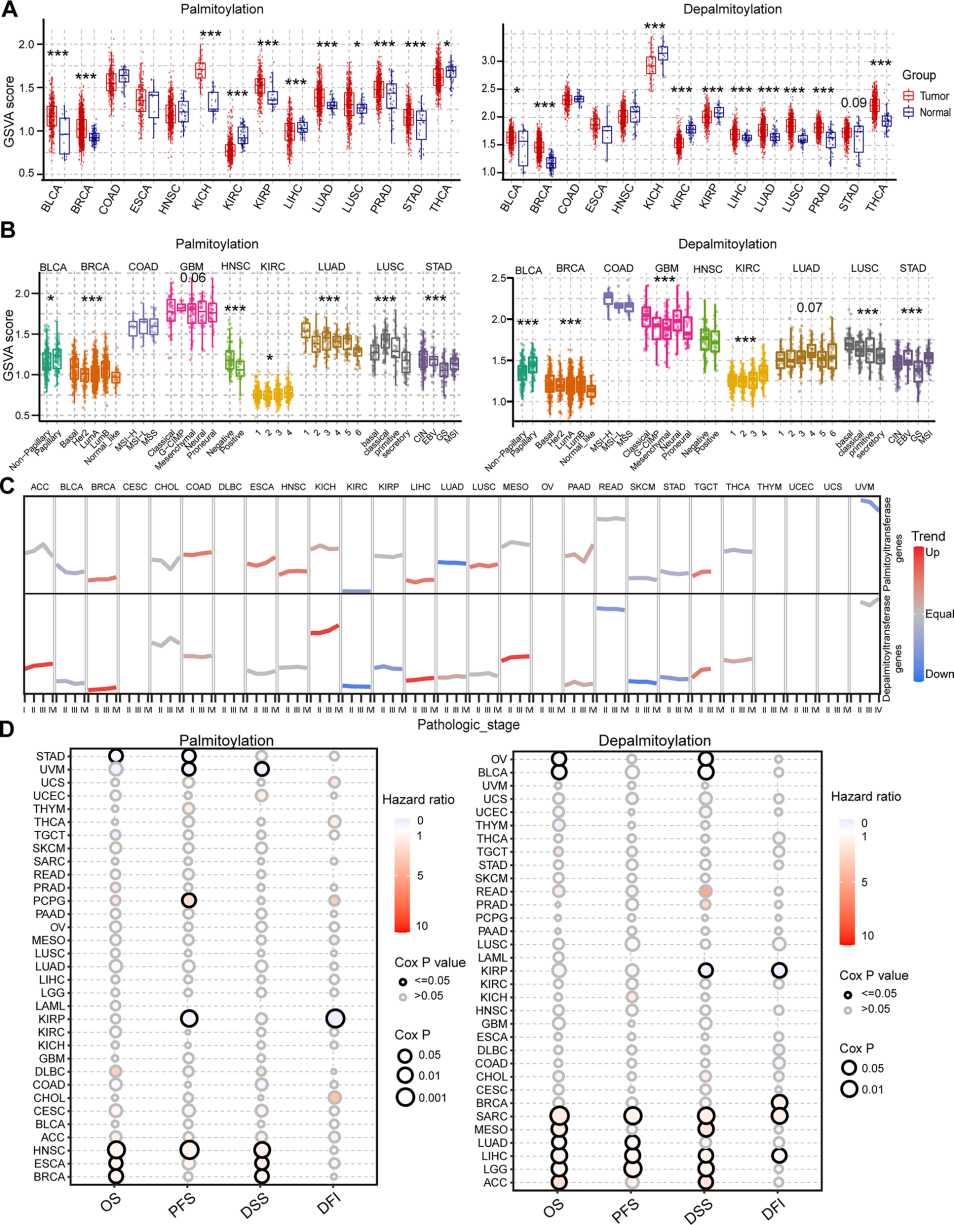
Dysregulated palmitoylation in human cancers. **A** Box plot showing the GSVA score of palmitoyl-acyltransferases (**Left**) and de-palmitoyl-acyltransferases (**Right**) genes between tumor and normal samples. **B** Box plot showing the GSVA score of palmitoyl-acyltransferases (**Left**) and de-palmitoyl-acyltransferases (**Right**) genes among subtypes in human cancers. **C** The trend of GSVA score of palmitoyl-acyltransferases (**Upper**) and de-palmitoyl-acyltransferases (**Lower**) genes between stages in human cancers. **D** Survival between high and low GSVA score of palmitoyl-acyltransferases (**Left**) and de-palmitoyl-acyltransferases (**Right**) genes in human cancers. *P < 0.05, **P < 0.01, ***P < 0.001

Further delving into the profiles of protein palmitoylation in human cancers, we performed GSVA in 14 cancer types using more than 10 pairs of tumor-normal samples from the TCGA database. The results revealed dysregulation of protein palmitoylation in these human cancers (Fig. 1A), implying its potential critical involvement in tumorigenesis. Additionally, the expression of palmitoylation-related genes demonstrated associations with cancer subtypes in most cancer types with available subtype information (Fig. 1B). Similarly, the expression patterns of these genes across different stages of cancer were linked to tumor progression in multiple cancer types (Fig. 1C).

Subsequently, we examined the association between the expression of palmitoylation-related genes and patient survival outcomes [5], including OS, PFS, DSS, and DFI, across the 33 cancer types. Our analysis revealed that dysregulation of palmitoylation profiles correlated with patient survival in several human cancers (Fig. 1D). These findings collectively highlight the dysregulation of protein palmitoylation in human cancers and its potential association with tumor progression.

### Dys-expression of palmitoylation-related genes across human cancers

**Fig. 2:**
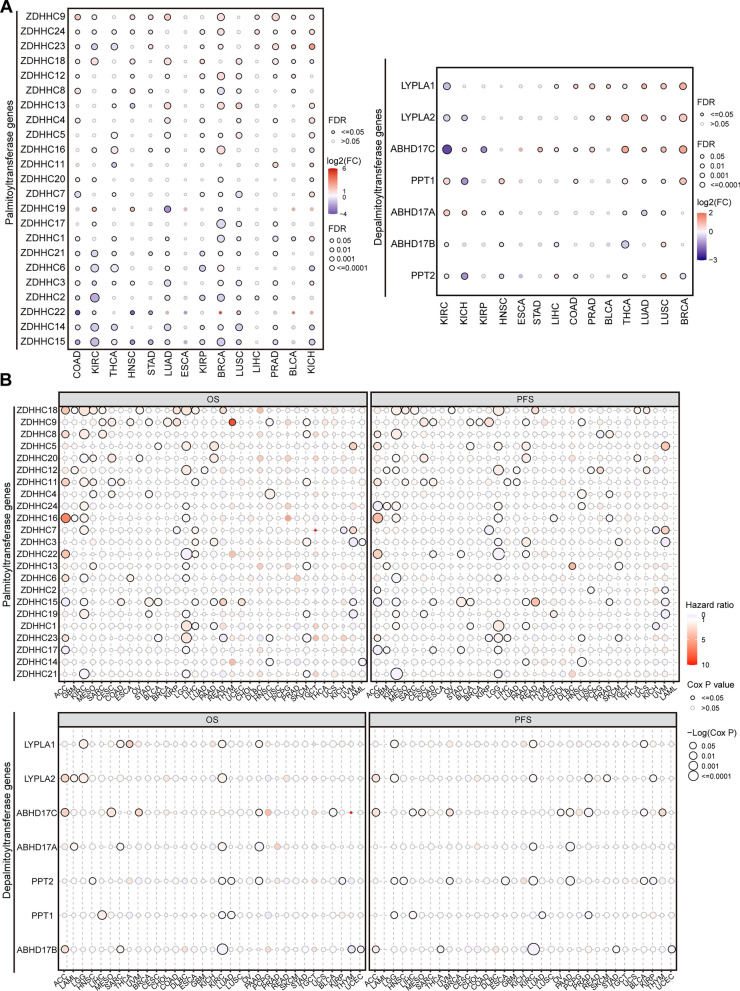
Dys-expression of palmitoylation-related genes across human cancers. **A** Bubble plot showing the differentially expressed palmitoyl-acyltransferases (**Left**) or de-palmitoyl-acyltransferases (**Right**) genes between tumor and normal samples in human cancers from the TCGA database. FC represents the fold change between tumor vs. normal. **B** Bubble plot showing survival difference between the high and low expression of palmitoyl-acyltransferases (**Upper**) or de-palmitoyl-acyltransferases (**Lower**) genes

In order to provide a comprehensive description of the expression landscape of palmitoylation in human cancers, we conducted a detailed analysis of the differential expression of each palmitoylation-related gene in normal and tumor tissues across the aforementioned 14 human cancer types. The results demonstrated that a majority of the palmitoylation-related genes exhibited differential expression patterns in various cancer types (Fig. 2A). Moreover, we observed a significant association between all palmitoylation-related genes and cancer subtypes in at least three human cancer types (Additional file 2: Fig. S2A). Notably, two specific genes, ZDHHC9 (a palmitoyl-acyltransferase) and ABHD17C (a de-palmitoyl-acyltransferase), displayed significant dysregulation across more than 10 human cancer types and exhibited correlation with cancer subtypes in seven human cancer types (Fig. 2A, Additional file 2: Fig. S2A). These findings suggest a potential crucial role for ZDHHC9 and ABHD17C in tumorigenesis.

We also assessed the relation between the expression of each individual palmitoylation-related gene and the prognosis of cancer patients, including OS, PFS, DSS, and DFI, across the 33 different human cancer types. The results indicated that all palmitoylation-related genes were associated with at least one survival prognosis in at least one cancer type (Fig. 2B, Additional file 2: Fig. S2B). Collectively, our analysis demonstrates the aberrant expression of palmitoylation-related genes in a variety of human cancers, as well as their association with cancer patient prognosis.

### Dys-expression of palmitoylation-related genes is mainly driven by DNA methylation alterations

The analysis of gene mutations is crucial for understanding tumorigenesis [36, 37]. Therefore, we collected mutation data from 10,234 samples across 33 cancer types sourced from the TCGA database. We specifically focused on seven types of deleterious mutations (Missense_Mutation, Nonsense_Mutation, Frame_Shift_Ins, Splice_Site, Frame_Shift_Del, In_Frame_Del, and In_Frame_Ins) to investigate their impact. Intriguingly, pan-cancer analysis revealed that palmitoylation-related genes had generally low mutation rates across most cancer types, with the exception of UCEC (Additional file 2: Fig. S3A). As expected, our survival analysis demonstrated that these mutations in palmitoylation-related genes influenced the prognosis of UCEC patients (Additional file 2: Fig. S3B), emphasizing their role in dysregulated palmitoylation, particularly in UCEC.

Considering the crucial role of CNV in gene expression regulation [38, 39], we also examined the relationship between the expression of palmitoylation-related genes and CNV. Interestingly, in half of the cancer types, none of the palmitoylation-related genes exhibited gains or losses at the CNV level (Additional file 2: Fig. S4A), although the correlations between the expression of palmitoylation-related genes and CNV of these genes were found (Additional file 2: Fig. S4B).

**Fig. 3:**
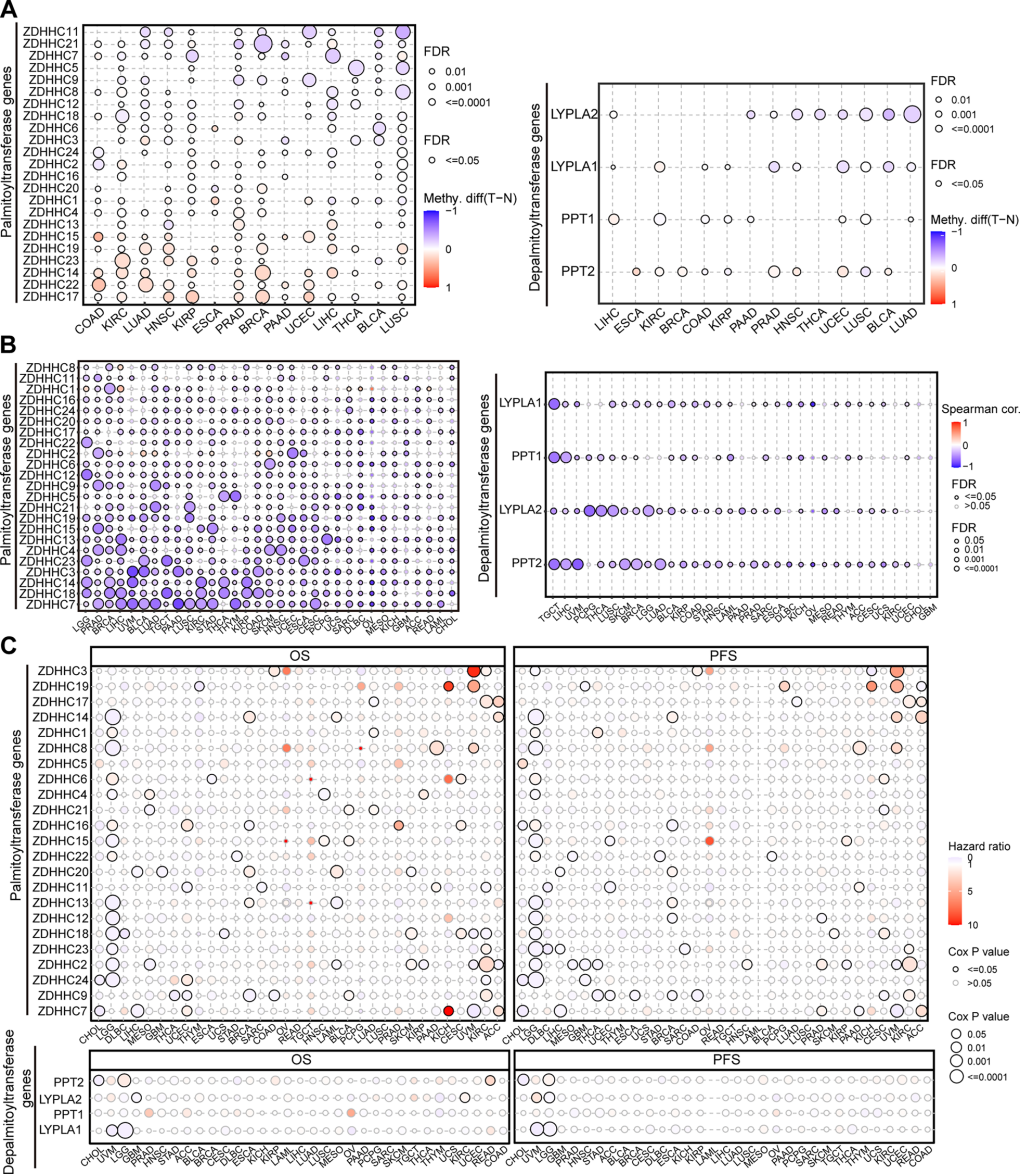
Dys-expression of palmitoylation-related genes is mainly driven by DNA methylation alterations. **A** Bubble plot showing the methylation difference between tumor and normal samples of palmitoyl-acyltransferases (**Left**) or de-palmitoyl-acyltransferases (**Right**) genes in human cancers. **B** Bubble plot showing the profile of correlations between methylation and mRNA expression of palmitoyl-acyltransferases (**Left**) or de-palmitoyl-acyltransferases (**Right**) genes in human cancers. **C** Bubble plot showing the survival difference between high and low methylation of palmitoyl-acyltransferases (**Upper**) or de-palmitoyl-acyltransferases (**Lower**) genes in human cancers

Moreover, given the ubiquitous presence of abnormal DNA methylation in cancer [36]. we analyzed whether dysregulation of palmitoylation-related genes could be attributed to DNA methylation alterations. We found that DNA methylation of all examined palmitoylation-related genes was abnormal in at least half of the analyzed cancer types (Fig. 3A). Moreover, the expression of these genes showed a negative correlation with their methylation levels (Fig. 3B). Importantly, methylation levels of palmitoylation-related genes significantly impacted the survival outcomes of patients in multiple cancer types, particularly LGG (Fig. 3C). In summary, our findings suggest that dysregulation of palmitoylation-related genes, driven by DNA methylation changes, is a notable aspect of tumorigenesis.

### Myc drives dysregulated palmitoylation

**Fig. 4:**
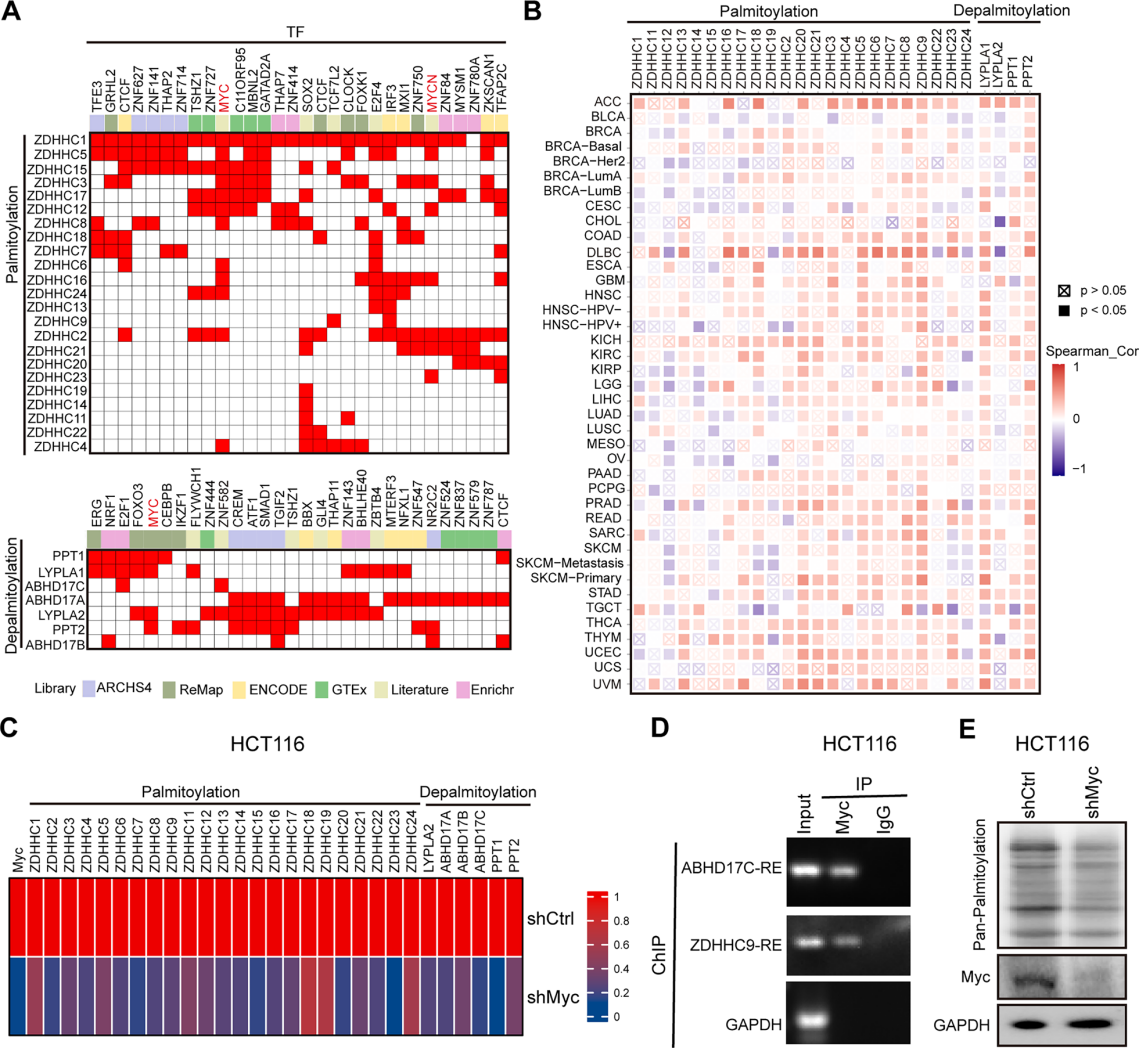
Myc drives dysregulated palmitoylation. **A** Clustergram showing the transcription factors that potentially regulate palmitoyl-acyltransferases (**Upper**) or de-palmitoyl-acyltransferases (**Lower**), based on the ChEA3 website. **B** The expression correlation between Myc and palmitoylation-related genes, based on TIMER2.0 website (http://timer.comp-genomics.org/timer/). **C** Heatmap showing the expression of palmitoyl-acyltransferases or de-palmitoyl-acyltransferases genes in Myc-depleted HCT116 cells. **D** Myc occupancy on the ZDHHC9 and ABHD17C promoters. ChIP was performed using the endogenous Myc antibody in HCT116 cells. PCR analysis was conducted on the endogenous promoters of ZDHHC9 and ABHD17C genes. **E** Global palmitoylation levels decrease upon Myc depletion in Myc-depleted HCT116 cells

Transcription factors play a crucial role in regulating gene expression by either activating or suppressing transcription processes [7, 23]. To investigate whether any specific transcription factor is involved in regulating the expression of palmitoylation-related genes, we conducted analysis using the ChEA3 website [40], which utilizes gene set libraries derived from published Chromatin Immunoprecipitation (ChIP)-seq data from various sources. Interestingly, our analysis revealed a significant positive association between Myc, a transcription factor frequently activated in cancer, and the majority of palmitoylation-related genes (Fig. 4A, B). This suggests that Myc may act as a regulator of dysregulated palmitoylation in human cancers.

To further investigate the regulatory role of Myc in the expression of palmitoylation-related genes, we performed depletion experiments of Myc in colorectal cancer cells, specifically HCT116, SW48 and HT29 cell lines. Consistent with our expectations, heatmap analysis revealed that depletion of Myc led to a significant decrease in the expression of most palmitoylation-related genes (Fig. 4C, Additional file 2: Fig. S5A). As part of our validation process, we discovered that Myc directly binds to the promoter of ZDHHC9 gene (Fig. 4D, Additional file 2: Fig. S5B, C). Additionally, we observed that depletion of Myc resulted in a decrease in global protein palmitoylation levels (Fig. 4D, Additional file 2: Fig. S5D). Collectively, these findings provide evidence that Myc drives the dysregulated profile of palmitoylation in human cancers.

### Dysregulated palmitoylation is associated with immune infiltration and immunotherapy response in human cancers

Immune infiltration has emerged as a clinically relevant biomarker, influencing prognosis and therapy response in cancer patients [24]. To explore the relationship between immune infiltration and palmitoylation in human cancers, we performed GSVA analysis focusing on palmitoylation and de-palmitoylation. Through Immune Cell Abundance Identifier [1], we evaluated the infiltration of 24 immune cell types. Our analysis revealed a significant association between dysregulated palmitoylation and different immune cell infiltrates across 33 human cancers, particularly in PPAD (Additional file 2: Fig. S6A, B). To verify these findings, we performed single-cell analysis, which demonstrated the expression of palmitoylation-related genes in immune cells to some extent in PAAD (Additional file 2: Fig. S6C–F).

**Fig. 5:**
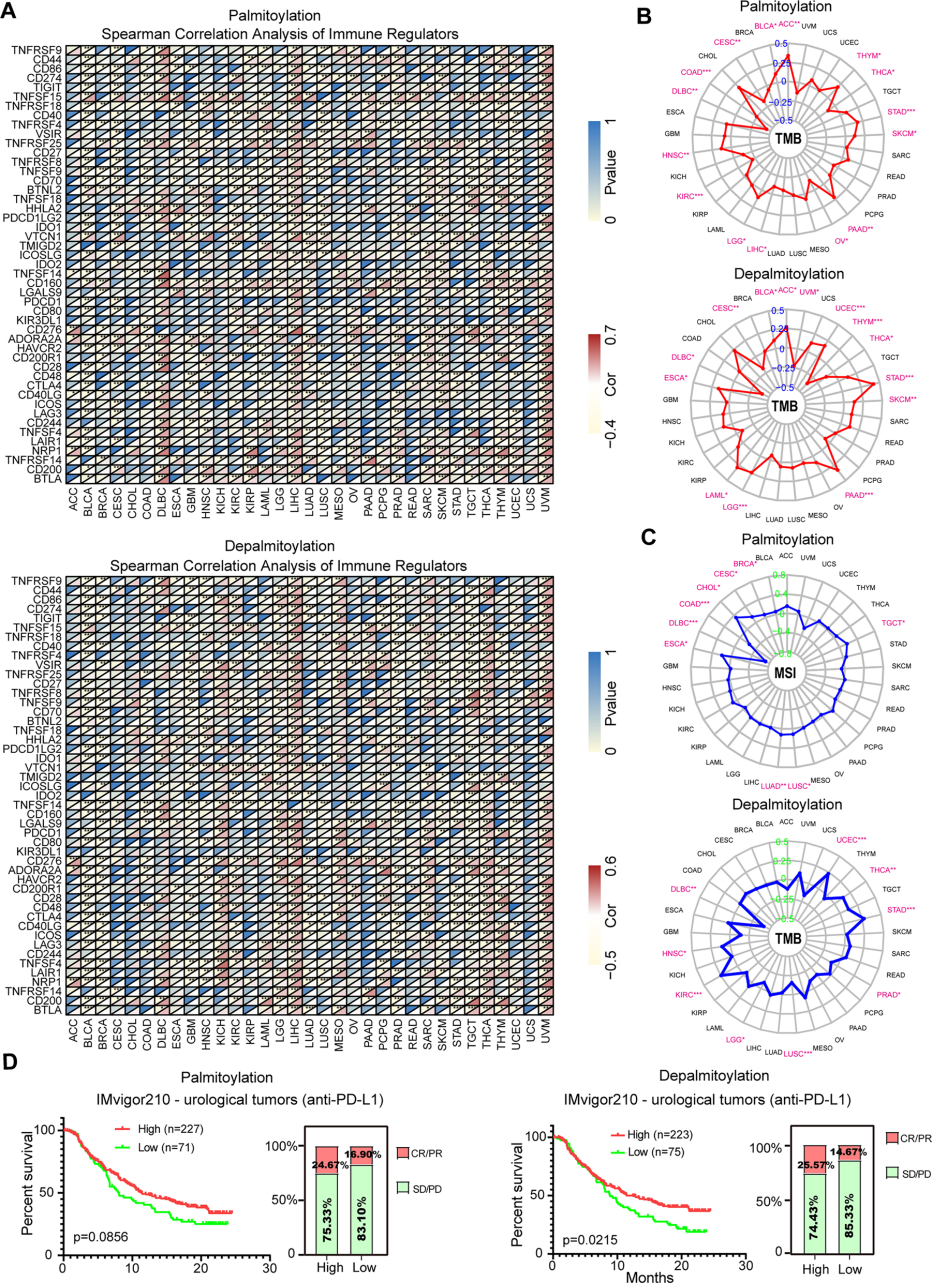
Dysregulated palmitoylation is associated with immunotherapy response in human cancers. **A** The spearman correlation heatmap shows the correlations between the palmitoylation-related genes expressions and the 47 types of immune regulators in pan-cancer. **B** Correlations between palmitoyl-acyltransferases (**Upper**) or de-palmitoyl-acyltransferases (**Lower**) gene expression and TMB in pan-cancer. **C** Correlations between palmitoyl-acyltransferases (**Upper**) or de-palmitoyl-acyltransferases (**Lower**) gene expression and MSI in pan-cancer. **D** Kaplan–Meier curves and response to anti-PD-1 therapy in Low- and High- palmitoyl-acyltransferases (**Left**) or de-palmitoyl-acyltransferases (**Right**) gene expression subgroups of IMvigor210 Cohort (Anti-PD-L1, Urological Tumors)

In addition, we investigated the relationship between dysregulated palmitoylation and 47 immune regulators in various cancer types. Remarkably, we observed a strong correlation between dysregulated palmitoylation and most immune regulators (Fig. 5A). Furthermore, we explored the predictive value of palmitoylation in assessing the effectiveness of immune checkpoint inhibitors (ICIs) by examining the correlation between dysregulated palmitoylation and tumor mutational burden (TMB) as well as microsatellite instability (MSI). Our results indicated a significant correlation between dysregulated palmitoylation and TMB/MSI across multiple cancer types (Fig. 5B, C). Interestingly, ZDHHC9 and ABHD17C demonstrate similar effects on TMB/MSI (Additional file 2: Fig. S7A, B). These findings suggest that analyzing palmitoylation levels could enable the identification of predictive markers for ICI effectiveness in specific cancer types.

Given the significant advancements in cancer immunotherapy, particularly the use of anti-PD-L1 antibodies[41], we further investigated the predictive role of the dysregulated palmitoylation in ICI-treated cancer cohorts, specifically focusing on patients with urological tumors. Remarkably, patients with low level of the palmitoylation/depalmitoylation exhibited worse survival rates and shorter survival times compared to those with high level (Fig. 5D). These findings demonstrate a correlation between dysregulated palmitoylation, immune infiltration, and immunotherapy response in human cancers.

### Palmitoylation regulates classical cancer-related pathways to affect tumorigenesis

**Fig. 6:**
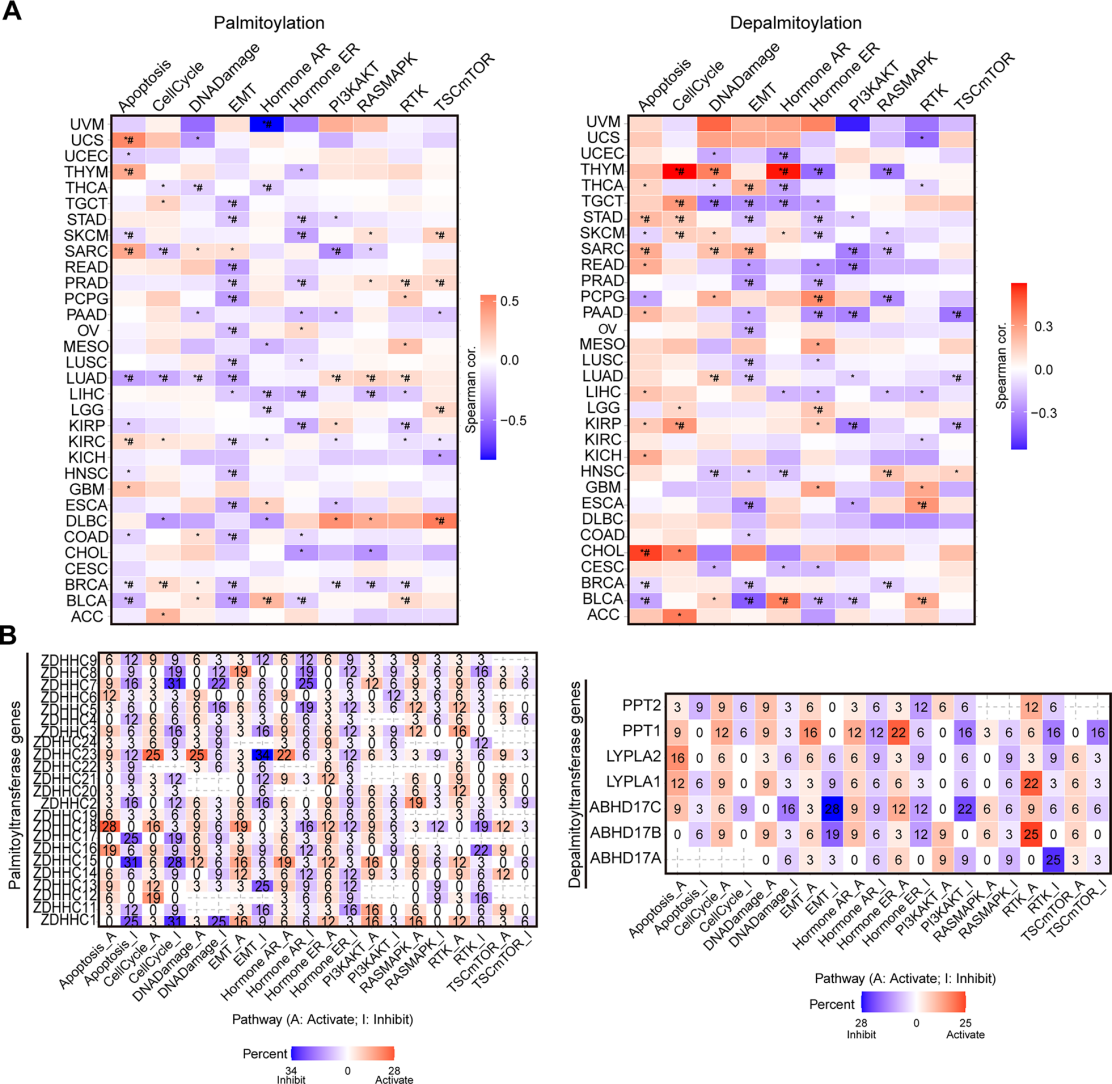
Palmitoylation regulates classical cancer-related pathways to affect tumorigenesis.** A** Heatmap summarizes the correlations between GSVA score of palmitoyl-acyltransferases (**Left**) or de-palmitoyl-acyltransferases (**Right**) genes and pathway activity among cancer types. **B** Heatmap showing the percentage of cancers in which palmitoyl-acyltransferases (**Left**) or de-palmitoyl-acyltransferases (**Right**) genes have effects (FDR <  = 0.05) on the pathway among 32 cancer types. The number in each cell indicates the percentage. A: Activate; I: Inhibit. *P < 0.05; ^#^FDR ≤ 0.05.

To gain further insights into palmitoylation-related signaling pathways, we utilized RPPA data from the TCPA database. We calculated pathway activity scores for 10 cancer-related pathways using RPPA data from 7,876 samples across 32 cancer types in the TCGA database. Interestingly, we observed associations between dysregulated palmitoylation and at least one cancer-related signaling pathway in each cancer type (Fig. 6A). These findings indicate that palmitoylation may influence classical cancer-related pathways and contribute to tumorigenesis.

To investigate the relationship between individual palmitoylation-related genes and these cancer-related pathways, we generated a heatmap displaying the percentage of cancers in which mRNA expression of a palmitoylation-related gene potentially affects pathway activity (Fig. 6B). For instance, palmitoyl-acyltransferase ZDHHC7 showed a negative association with DNA damage and hormone AR pathways in seven and eight cancer types, respectively (Fig. 6B). Similarly, palmitoyl-acyltransferase ZDHHC23 displayed a positive association with DNA damage and mTOR pathways in eight and three cancer types, respectively. These findings highlight the potential role of ZDHHC7 and ZDHHC23 in modulating cancer-related pathways.

**Fig. 7:**
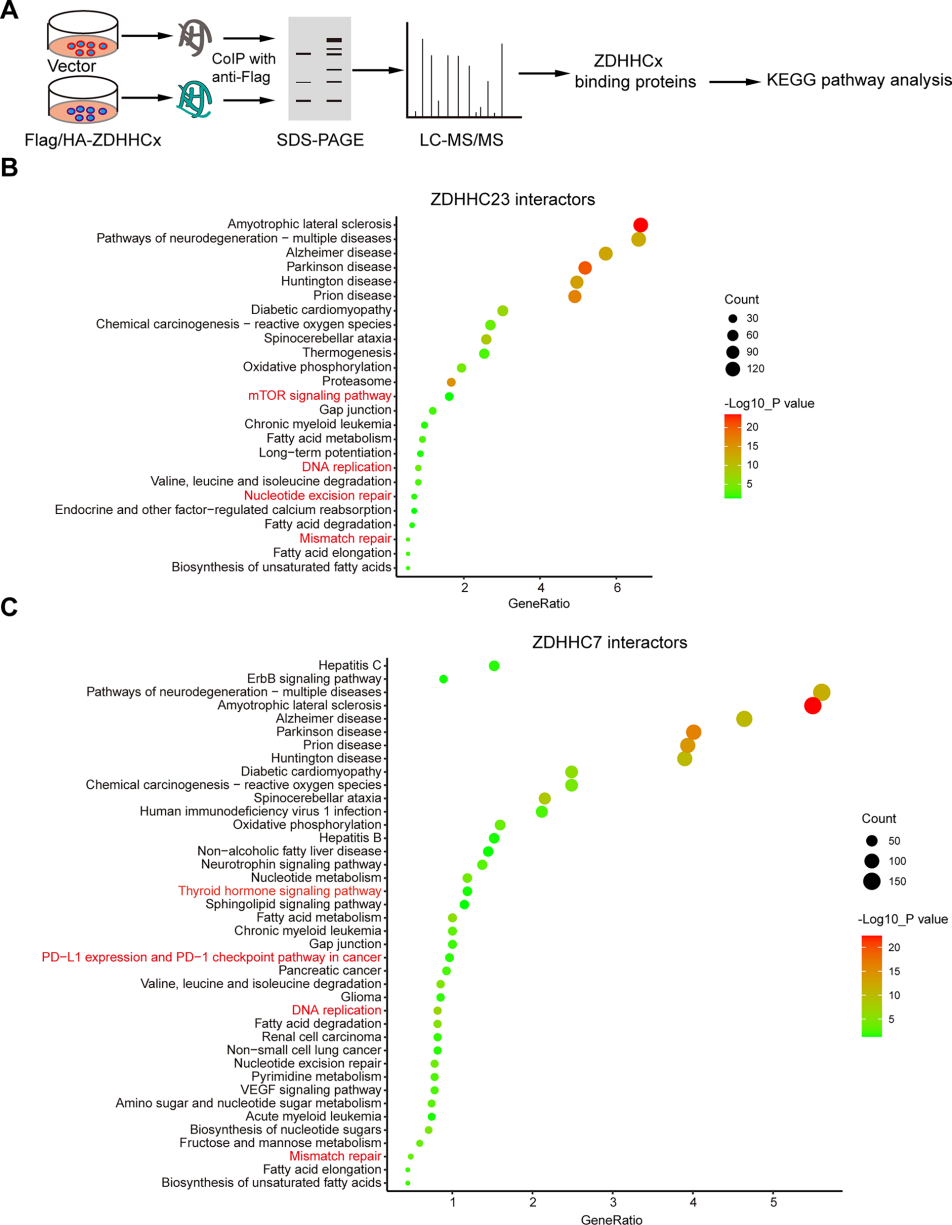
Plmitoylacyltransferase ZDHHC7 and ZDHHC23 are associated with classical cancer-related pathways and immune pathways.** A** Schematic diagram showing the experimental design. Flag or HA-labeled ZDHHC7 and ZDHHC23 were transfected into HEK293T cells, and then the epitope-labeled proteins were immunoprecipitated for mass spectrometry analysis. The ZDHHC7- and ZDHHC23-associated proteins were then subjected to KEGG analysis. **B**, **C** Bubble plot showing the ZDHHC7- (**B**) and ZDHHC23 (**C**)-associated pathways

To strengthen our analysis, we performed experimental verification using exogenously expressed ZDHHC7 and ZDHHC23 in HEK293T cells. Immunoprecipitation of the epitope-labeled proteins (Flag or HA-tagged) followed by mass spectrometry analysis allowed us to identify ZDHHC7- and ZDHHC23-associated proteins. Subsequently, Kyoto Encyclopedia of Genes and Genomes (KEGG) pathway analysis was conducted on these associations (Fig. 7A). Consistent with our previous analysis, we observed significant correlations between ZDHHC7 or ZDHHC23 and pathways related to mTOR, DNA repair, hormone signaling, and immune response (Fig. 7B, C).Taken together, our data suggest that palmitoylation plays a role in the regulation of classical cancer-related pathways, thereby impacting tumorigenesis.

### Potential small molecules regulating palmitoylation are identified

To identify potential drugs that can regulate palmitoylation, we gathered IC50 values for 481 small molecules and their corresponding mRNA gene expressions from the CTRP database. By conducting a correlation analysis between the mRNA expression of each palmitoylation-related gene and the drug IC50 values, we identified a range of small molecules associated with these genes (Additional file 2: Fig. S8A). Specifically, 19 small molecules emerged as potential regulators of palmitoylation (Additional file 2: Fig. S8B).

**Fig. 8:**
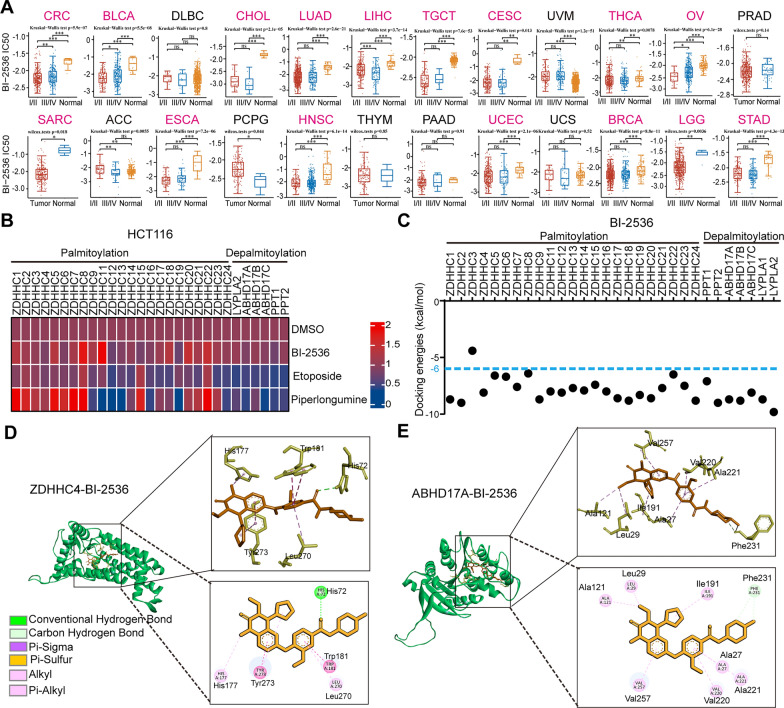
Potential small molecules regulating palmitoylation are identified. **A** Pan-cancer boxplot of BI-2536 IC50 values in normal tissue and stage tumors. **B** Heatmap of palmitoyl-acyltransferases and de-palmitoyl-acyltransferases gene expression in HCT116 cells treated with BI-2536 (5 μM), etoposide (1 μM), and piperlongumine (10 μM) after 24 h. **C** Scatter plot of docking energies between BI-2536 and palmitoylation-related proteins. **D**, **E** Docking results of ZDHHC4 (**D**) and ABHD17A (**E**) proteins with small molecular compound BI-2536

To validate these findings, we examined data from the GDSC and TCGA databases. Among the 19 small molecules, only three (BI-2536, etoposide, and piperlongumine) matched the available data. We performed pan-cancer susceptibility analysis for these three small molecules and found that they significantly inhibited multiple types of cancer (Fig. 8A, Additional file 2: Fig. S8C, D). Notably, treatment with BI-2536 or piperlongumine resulted in decreased expression of some palmitoylation-related genes in HCT116, SW48 and HT29 cells (Fig. 8B, Additional file 2: Fig. S9), suggesting that these small molecules may regulate palmitoylation by modulating the expression of these genes.

To analyze potential targeted small molecules, we focused on the three aforementioned compounds: BI-2536, etoposide, and piperlongumine. Using AlphaFold2, we conducted protein structure predictions and performed molecular docking calculations with Autodock Vina. Our predictions revealed that all ZDHHC enzymes possess conserved 4 alpha-helical bundles, with the enzymatic regions situated within this region (Additional file 1: Table S5). Through analysis of protein–ligand binding energies and intermolecular interactions, we observed that, except for ZDHHC3, all proteins exhibited favorable binding affinity to BI-2536 (binding energy below -6.0 kcal/mol), with most forming strong hydrogen bond interactions with the compound (Fig. 8C, Additional file 1: Table S5). Etoposide and piperlongumine also displayed favorable binding energies with these proteins (Additional file 2: Fig. S10A).

The binding pockets for the small molecules within the ZDHHC family are located in the hydrophobic interior of the proteins (Fig. 8D, Additional file 2: Fig. S10B–E), while the binding pockets for other proteins (PPT1/2, LYPLA1/2, ABHD17A/B/C) are situated on the protein surface (Fig. 8E, Additional file 2: Fig. S10F, G), as opposed to the enzymatic pockets near the 4 alpha-helical bundles of the ZDHHC enzymes (Fig. 8D, Additional file 2: Fig. S10B–E). Despite belonging to different protein families and exhibiting structural variations, these molecules can effectively regulate the expression of palmitoylation-related genes or bind to palmitoylation-related proteins, making them promising drug candidates for targeting these proteins.

## Discussion

In this study, we have provided a comprehensive overview of the landscape of palmitoylation in human cancers, shedding light on its pivotal role in tumorigenesis. We observed that depletion of Myc, a known transcription factor, leads to alterations in the expression profile of palmitoylation-related genes, further supporting the involvement of Myc in the regulation of palmitoylation. Additionally, we found a correlation between dysregulated palmitoylation and immune infiltration as well as classical cancer-related pathways, providing valuable insights into the mechanisms by which palmitoylation impacts tumorigenesis.

While previous studies have associated palmitoylation with tumorigenesis [18, 42, 43], a comprehensive understanding of the overall landscape of palmitoylation across various human cancers has been lacking. To address this, we utilized palmitoylation-related genes as surrogates for global palmitoylation levels in cancer and conducted pan-cancer analysis. Our results revealed dysregulated palmitoylation patterns in human cancers. Importantly, we also discovered that these dysregulations in palmitoylation are associated with alterations in DNA methylation, highlighting the significant role of epigenetics in modulating palmitoylation.

Oncogenic Myc, a prominent transcription factor, has been extensively studied for its role in promoting tumorigenesis through the regulation of diverse biological processes [44–47]. However, the specific relationship between Myc and palmitoylation has not been previously reported. In our study, we made a novel discovery that depletion of Myc results in a substantial decrease in global palmitoylation levels. This finding strongly suggests that Myc has the capability to regulate the process of palmitoylation, adding a new dimension to our understanding of Myc’s role in tumorigenesis.

Immune infiltration plays a crucial role in tumor development, prognosis, and response to immunotherapy. Recent research has indicated that the palmitoylation of proteins such as PDL1 and IFNGR1 can impact the levels of CD8^+^ T cell infiltration and influence the effectiveness of immunotherapy in human cancer [19, 32]. In our study, we conducted a comprehensive pan-cancer analysis to explore the landscape of palmitoylation and its relationship with immune infiltration. Through our analysis, we were able to gain a better understanding of the impact of palmitoylation on immune infiltration in various cancer types. Our findings suggest that modifying the palmitoylation status of certain proteins could be a viable strategy in improving the immune response in immunotolerant patients. This knowledge may offer potential therapeutic targets for enhancing the efficacy of immunotherapy interventions. Overall, our study contributes to the growing understanding of the role of palmitoylation in immune infiltration and its implications for cancer treatment.

The overactivation or inactivation of signaling pathways is responsible for the uncontrolled proliferation and survival of tumor cells [6, 35, 48]. Recent studies have shown that palmitoylation plays a role in regulating cancer-related pathways, influencing tumor initiation and progression [35, 48]. Consistently, our research also found a significant association between palmitoylation and classical cancer-related pathways in human cancers. In addition, we constructed a comprehensive landscape of palmitoylation and its relationship with these pathways, which enhances our understanding of the mechanisms underlying tumorigenesis. Of particular importance, our study identified three drugs—BI-2536, etoposide, and piperlongumine—that have the potential to regulate palmitoylation by targeting specific gene expression or enzymes involved in the process. Notably, these drugs have demonstrated anti-tumor potential in previous studies [49–51]. It is worth mentioning that BI-2536, a potent inhibitor of PLK1, has been reported to promote tumor growth by stabilizing Myc. Consequently, it is likely that BI-2536 targets palmitoylation by inhibiting the expression of Myc.

## Limitations

Although our findings in this study have important clinical implications, there are still some limitations that need to be addressed. Firstly, we found that palmitoylation is associated with TMB and MSI, but the relationship remains unclear. Secondly, while we have identified potential small molecule drugs that may regulate palmitoylation, specifically BI-2536, etoposide, and piperamine, it is important to note that further research is necessary to validate these findings. Our future research will be dedicated to conducting additional experiments that specifically address these questions and aim to provide more conclusive evidence.

## Conclusions

In summary, our data provide a comprehensive overview of palmitoylation in various human cancers, highlight the role of DNA methylation alterations and Myc in palmitoylation regulation, and elucidate the intricate relationship between palmitoylation and cancer-related pathways. Notably, our identification of three drugs with the potential to regulate palmitoylation offers new possibilities for cancer therapy.

### Supplementary Information

**Additional file 1: Table S1.** List of primer and shRNA sequences used in this study. **Table S2.** List of TCGA cancer types.**Table S3.** List of antibodies used in this study. **Table S4.** List of palmitoylation-related genes. **Table S5.** Docking information of BI-2536 and palmitoylation-associated proteins.

**Additional file 2: Fig. S1.** Expression of most palmitoylation-related genes is equal in various normal tissues. **Fig. S2.** Expression of palmitoylation-related genes is associated with cancer subtype and patient survival. **Fig. S3.** Mutation landscape of palmitoylation-related genes in human cancers. **Fig. S4.** CNV landscape of palmitoylation-related genes in human cancers. **Fig. S5.** Myc regulates palmitoylation. **Fig. S6.** Dysregulated palmitoylation is associated with immune infiltration in human cancers. **Fig. S7.** The expression levels of ZDHHC9 and ABHD17C genes are correlated with TMB and MSI. **Fig. S8.** Etoposide and piperlongumine are potential small molecules for regulating palmitoylation. **Fig. S9.** BI-2536, etoposide and piperlongumine regulate the expression of palmitoylation-related genes.** Fig. S10.** BI-2536, etoposide and piperlongumine are potential small molecules for targeting palmitoylation-related proteins.

## Data Availability

All data that support the findings in this study are available from the corresponding author upon reasonable request.

## Abbreviations

c-Myc: MYC proto-oncogene, bHLH transcription factor
ZDHHC7: Zinc finger DHHC-type palmitoyltransferase 7
ZDHHC23: Zinc finger DHHC-type palmitoyltransferase 23
mTOR: Mechanistic target of rapamycin kinase
CNV: Copy number variant
TCGA: The cancer genome atlas
GTEx: Genotype-tissue expression
ImmuCellAI: Immune cell abundance identifier
CTRP: Cancer therapeutics response portal
GDSC: Genomics of drug sensitivity
GSCA: Gene set cancer analysis
GSVA: Gene set variation analysis
FDR: False discovery rate
OS: Overall survival
PFS: Progression-free survival
DSS: Disease-specific survival
DFI: Disease-free interval
RT-qPCR: Real-time quantitative PCR
ABE: Acyl-biotin exchange
LYPLA1: Lysophospholipase 1
LYPLA2: Lysophospholipase 2
PPT1: Palmitoyl-protein thioesterase 1
PPT2: Palmitoyl-protein thioesterase 2
PDB: Protein data bank
ZDHHC: Zinc finger DHHC-type containing
β-catenin: Catenin beta 1
PD-L1: CD274 molecule
IFNGR1: Interferon gamma receptor 1
ZDHHC18: Zinc finger DHHC-type palmitoyltransferase 18
ZDHHC19: Zinc finger DHHC-type palmitoyltransferase 19
ZDHHC9: Zinc finger DHHC-type palmitoyltransferase 9
ABHD17C: Abhydrolase domain containing 17C, depalmitoylase
ABHD17A: Abhydrolase domain containing 17A, depalmitoylase
ABHD17B: Abhydrolase domain containing 17B, depalmitoylase
ChIP: Chromatin Immunoprecipitation
ICIs: Immune checkpoint inhibitors
TMB: Tumor mutational burden
MSI: Microsatellite instability
