# Supplementary material for: Palmitoylation landscapes across human cancers reveal a role of palmitoylation in tumorigenesis
Source: J Transl Med. 2023 Nov 17;21:826. doi: 10.1186/s12967-023-04611-8 (PMC10655258; doi:10.1186/s12967-023-04611-8)
Supplement: Supplementary file 1 — Additional file 1: Table S1. List of primer and shRNA sequences used in this study. Table S2. List of TCGA cancer types.Table S3. List of antibodies used in this study. Table S4. List of palmitoylation-related genes. Table S5. Docking information of BI-2536 and palmitoylation-associated proteins. [file 12967_2023_4611_MOESM1_ESM.docx]

**Additional file 1**

**Table S1 List of primer and shRNA sequences used in this study.**

**qPCR primers**

| Amplicons | Forward Primer (5’>3’) | Reverse Primer (5’>3’) |
| --- | --- | --- |
| *ZDHHC1* | GTGCGGGACAAGAGCTATG | AGTTGCAGTGCAGGTCTTCAA |
| *ZDHHC2* | TCCCGGTGGTGTTCATCAC | CAACTTGTTCGCCAGTGTTTTC |
| *ZDHHC3* | CCACTTCCGAAACATTGAGCG | CCACAGCCGTCACGGATAAA |
| *ZDHHC4* | GTACACCTGGGAAGTATTTGGC | CTAGCAGCAGATAGGGCAGAA |
| *ZDHHC5* | CACCTGCCGCTTTTACCGT | CGGCGACCAATACAGTTATTCAC |
| *ZDHHC6* | GTTGTGGTATTGGCCCTTACA | AAAGCCCGGACCGACAAAC |
| *ZDHHC7* | CTGACCGGGTCTGGTTCATC | CATGACGAAAGTCACCACGAA |
| *ZDHHC8* | CTCAAACCCGCCAAGTACATC | ACACAGCTCGTGTCAACCAC |
| *ZDHHC9* | CCTGGGTGGGGAATTGTGTT | ACGACGGACCAGAGTGTAAAG |
| *ZDHHC11* | GGTGCAGACCCTGATAGTCG | GCACGTATGGATCTTTCCTCAC |
| *ZDHHC12* | CTCCTCGTCTCGCACCTCTA | GATAGGCGATGCGGTGTGAG |
| *ZDHHC13* | AGGAAGCCATTAAGGTCACTCC | GCCAAAACCTATGCACCGTC |
| *ZDHHC14* | ACAAGTTCTTCTGTAACGGGAGG | GAGTCCGCTAGTGACCAGGA |
| *ZDHHC15* | GGTGCCAGTGCTCGTTATTGT | AAGACGTAGGCATAGTAGGACC |
| *ZDHHC16* | CTGGGTGCCCTAACTGTATGG | CTTGTTGATGTGCCTTTCGATG |
| *ZDHHC17* | GGCCCGGATGAGTACGATAC | TCCAAGAGGTTCACCATATCCA |
| *ZDHHC18* | CACCCCGAACCTCACACTG | TGAAGGCCGTCAGGAATGAGA |
| *ZDHHC19* | CACTGGGACCCAAGTACATGG | ATTGGAGGGTGCAGATTCGG |
| *ZDHHC20* | TTCGTGGTCGTCTGGTCCTA | AGGTAAACAACGGTCTTTCCATT |
| *ZDHHC21* | ACTGAACTTCTTACTTGCTACGC | AACAAAGAGGTCCAAATTACGCT |
| *ZDHHC22* | CCCTGGCGCAAGAACTTACAA | CTTCCGACATTGAACATGGGG |
| *ZDHHC23* | CAGAAGGGCAGTATGAAGCCT | CCATTCCGATCTATGTACTCGCA |
| *ZDHHC24* | CTGGCACAGTTTGCCTTGG | CAGGGACCCAGGTCATAGGAG |
| *PPT1* | AGCCGAATACTGGCATGACC | TTGATACCCCGCTCCTGATTT |
| *PPT2* | ACAGTGCTCGATCTCTTCGAT | CAGCCTCTCGGAACCCTTG |
| *LYPLA1* | CACTCAGTTGCTGGCTTCCACT | CCGTAAGAGAACCAAACATCAGG |
| *LYPLA2* | CCTCACGTCAAGTACATCTGT | GACGATTCGATTGGCAGGGAT |
| *ABHD17A* | GGAACCTCTATGCCGACATCG | GCCCGTACAGGATGATGCT |
| *ABHD17B* | TATGGCATTCGCCCTGAAAAT | GAGCAGCAAGATCCACAGACG |
| *ABHD17C* | ACTGTCCCCACGGTAGACTT | CACACGCAAACCAGACATCAG |
| *c-Myc* | TCCCTCCACTCGGAAGGAC | CTGGTGCATTTTCGGTTGTTG |
| *GAPDH* | GGAGCGAGATCCCTCCAAAAT | GGCTGTTGTCATACTTCTCATGG |

**shRNA sequences**

| shRNA | Sequences (5’>3’) |
| --- | --- |
| shMyc | CAGTTGAAACACAAACTTGAA |
| shCtrl | TTCTCCGAACGTGTCACGT |

**Primers for ChIP**

| Gene Promoter | Forward Primer (5’>3’) | Reverse Primer (5’>3’) |
| --- | --- | --- |
| *ZDHHC9* | GCACCGCCCTTCTCAACTCG | TCTCGAGGCCCGCCCTGACT |
| *ABHD17C* | GTTCATGACTAAGACCACTG | GTATAGGTAGCAACAGCGTT |
| *GAPDH* | GGAGCGAGATCCCTCCAAAAT | GGCTGTTGTCATACTTCTCATGG |

**Table S2 List of TCGA cancer types.**

| **Project (Abbreviations)** | **Cancer Type** | **Paired** | **Tumor** | **Normal** |
| --- | --- | --- | --- | --- |
|  |  |  |  |  |
|  |  |  |  |  |
| **ACC** | Adrenocortical Carcinoma | 0 | 79 | 0 |
| **BLCA** | Bladder Urothelial Carcinoma | 19 | 408 | 19 |
| **BRCA** | Breast Invasive Carcinoma | 114 | 1104 | 114 |
| **CESC** | Cervical Squamous Cell Carcinoma and Endocervical Adenocarcinoma | 3 | 307 | 3 |
| **CHOL** | Cholangiocarcinoma | 9 | 36 | 9 |
| **COAD** | Colon Adenocarcinoma | 26 | 288 | 41 |
| **DLBC** | Lymphoid Neoplasm Diffuse Large  B-cell Lymphoma | 0 | 48 | 0 |
| **ESCA** | Esophageal Carcinoma | 11 | 185 | 11 |
| **GBM** | Glioblastoma Multiforme | 0 | 169 | 5 |
| **HNSC** | Head and Neck Squamous Cell Carcinoma | 43 | 522 | 44 |
| **KIRC** | Kidney Renal Clear Cell Carcinoma | 72 | 534 | 72 |
| **KIRP** | Kidney Renal Papillary Cell Carcinoma | 32 | 291 | 32 |
| **LAML** | Acute Myeloid Leukemia | 0 | 173 | 0 |
| **LGG** | Brain Lower Grade Glioma | 0 | 534 | 0 |
| **LIHC** | Hepatocellular Carcinoma | 50 | 374 | 50 |
| **LUAD** | Lung Adenocarcinoma | 58 | 517 | 59 |
| **LUSC** | Lung Squamous Cell Carcinoma | 51 | 503 | 51 |
| **MESO** | Mesothelioma | 0 | 87 | 0 |
| **OV** | Ovarian Serous Cystadenocarcinoma | 0 | 309 | 0 |
| **PAAD** | Pancreatic Adenocarcinoma | 4 | 179 | 4 |
| **PCPG** | Pheochromocytoma and Paraganglioma | 3 | 184 | 3 |
| **PRAD** | Prostate Adenocarcinoma | 52 | 498 | 52 |
| **READ** | Rectum adenocarcinoma | 6 | 95 | 10 |
| **SARC** | Sarcoma | 2 | 263 | 2 |
| **SKCM** | Skin Cutaneous Melanoma | 1 | 473 | 1 |
| **STAD** | Stomach Adenocarcinoma | 32 | 415 | 35 |
| **TGCT** | Testicular Germ Cell Tumors | 0 | 156 | 0 |
| **THCA** | Thyroid Carcinoma | 59 | 513 | 59 |
| **THYM** | Thymoma | 2 | 120 | 2 |
| **UCEC** | Uterine Corpus Endometrial Carcinoma | 7 | 177 | 24 |
| **UCS** | Uterine Carcinosarcoma | 0 | 57 | 0 |
| **UVM** | Uveal Melanoma | 0 | 80 | 0 |

**Table S3 List of antibodies used in this study.**

| Protein | Company | Catalog No. | Source | Dilution |
| --- | --- | --- | --- | --- |
| GAPDH | Abbkine | ABP50163 | Rabbit polyclonal antibody | 1:2000 (WB) |
| c-Myc | Santa Cruz | 9E10 | Mouse monoclonal antibody | 1:200 (WB) |
| HA | Sigma | H6908 | Rabbit monoclonal antibody | 1:5000 (WB) |
| HA | Abclonal | AE008 | Mouse monoclonal antibody | 1:2000 (WB) |
| Flag | Proteintech | 20543-1-AP | Rabbit polyclonal antibody | 1:3000 (WB) |

**Table S4 List of palmitoylation-related genes.**

**Palmitoylacyltransferase genes**

| Gene |  |
| --- | --- |
|  | ID |
|  |  |
| ZDHHC1 | 29800 |
| ZDHHC2 | 51201 |
| ZDHHC3 | 51304 |
| ZDHHC4 | 55146 |
| ZDHHC5 | 25921 |
| ZDHHC6 | 64429 |
| ZDHHC7 | 55625 |
| ZDHHC8 | 29801 |
| ZDHHC9 | 51114 |
| ZDHHC11 | 79844 |
| ZDHHC12 | 84885 |
| ZDHHC13 | 54503 |
| ZDHHC14 | 79683 |
| ZDHHC15 | 158866 |
| ZDHHC16 | 84287 |
| ZDHHC17 | 23390 |
| ZDHHC18 | 84243 |
| ZDHHC19 | 131540 |
| ZDHHC20 | 253832 |
| ZDHHC21 | 340481 |
| ZDHHC22 | 283576 |
| ZDHHC23 | 254887 |
| ZDHHC24 | 254359 |

**de-palmitoylacyltransferase genes**

| Gene |  |
| --- | --- |
|  | ID |
|  |  |
| PPT1 | 5538 |
| PPT2 | 9374 |
| LYPLA1 | 10434 |
| LYPLA2 | 11313 |
| ABHD17A | 81926 |
| ABHD17B | 51104 |
| ABHD17C | 58489 |

**Table S5 Docking information of BI-2536 and palmitoylation-associated proteins.**

| **Protein** | **Enzyme active region （AA)** | **Binding energy (kcal/mol)** | **Ligand interaction** | |
| --- | --- | --- | --- | --- |
|  |  |  | **Hydrogen Bond** | **Non-hydrogen bond** |
| ZDHHC1 | 129-285 | -8.7 | GLY381;LEU260 | LYS165;TRP166;TRP380 |
| ZDHHC2 | 123-249 | -9 | SER224 | TRP159;LEU228;HIS155 |
| ZDHHC3 | 126-243 | -4.4 | NONE | LEU219;LEU226;LEU54;TRP159 |
| ZDHHC4 | 144-294 | -8.1 | HIS72 | HIS177;TYR273;TRP181;LEU270 |
| ZDHHC5 | 99-224 | -6.6 | GLY228;ARG213;ARG226 | VAL229;ARG215 |
| ZDHHC6 | 94-244 | -6.7 | HIS128 | PRO130;TRP131;PHE144;HIS127;CYS129;LEU223;ILE226;SER237 |
| ZDHHC7 | 129-258 | -7.6 | GLU141;GLU258 | ARG142;PRO140;ILE138 |
| ZDHHC8 | 99-224 | -6.4 | ARG215;PHE225;GLY228;ARG226;LYS752 | NONE |
| ZDHHC9 | 134-263 | -8.7 | NONE | TRP171;LEU242;ILE188;VAL239;PHE187;LEU191 |
| ZDHHC11 | 120-275 | -8 | NONE | VAL44;LEU243;LEU246;HIS250;TRP157;LEU251 |
| ZDHHC12 | 93-218 | -8.1 | CYS127;TYR145 | TRP129;HIS125;HIS201;LEU197 |
| ZDHHC13 | 421-559 | -7.7 | ARG425;LYS435;LYS573 | LEU440;HIS553;LEU558 |
| ZDHHC14 | 160-289 | -7.9 | NONE | LEU70;VAL257;PHE260 |
| ZDHHC15 | 125-249 | -7.4 | TRP161 | LEU230;PHE223;LEU227;PHE177;ILE28 |
| ZDHHC16 | 151-305 | -8 | SER155 | LYS164;VAL154;ARG167;GLU307;ILE303 |
| ZDHHC17 | 434-570 | -8.6 | SER450;LYS451;HIS452;VAL448 | LEU435;LYS446; |
| ZDHHC18 | 187-316 | -8.8 | MET188;SER316;ARG321;GLU324 | LYS314;LYS192;ARG201;ARG204;VAL189;TRP317 |
| ZDHHC19 | 108-232 | -8.3 | CYS142 | HIS140;LEU211;LEU208;TPR144;PRO207 |
| ZDHHC20 | 121-247 | -8.6 | TRP158;SER178;SER223 | VAL224;LEU227;PRO157 |
| ZDHHC21 | 85-218 | -7.7 | NONE | LEU150;ILE188;LEU191;PHE154;TYR157;TYR158 |
| ZDHHC22 | 83-222 | -6.5 | PHE113;ALA194;CYS137 | ILE18;TYR14;ALA198;ILE195 |
| ZDHHC23 | 254-388 | -7.5 | TPR291;ILE361 | ILE360;ILE368;MET365 |
| ZDHHC24 | 91-234 | -8.8 | LEU209 | ALA146;CYS210;LEU142;ALA26;LEU65;LEU66;LEU62;VAL149;LEU150 |
| PPT1 | 34-289 | -7.1 | GLY42;ASN47 | ILE54;LEU292 |
| PPT2 | 38-283 | -9 | PHE46;SER111;TYR148 | ASP47;TYR50;HIS283 |
| ABHD17A | 108-235 | -8.7 | PHE231 | LEU29;ALA121;ALA27;ILE191;ALA221;VAL220;VAL257 |
| ABHD17B | 133-286 | -8.8 | ASN100 | LEU9;LEU26;PHE13;ALA201;ALA213;ALA23;ALA27; |
| ABHD17C | 129-249 | -8.1 | ASN141;SER211;ILE212 | VAL278;HIS305 |
| LYPLA1 | 8-226 | -8.7 | NONE | LEU63;LEU78;TRP145;LEU176;LEU30;HIS208;VAL177 |
| LYPLA2 | 11-228 | -9.8 | SER122;TRP148;PHE183;HIS210; | LEU81;LEU33,LEU78;LEU66;MET178;HIS152 |
