# Supplementary material for: Palmitoylation landscapes across human cancers reveal a role of palmitoylation in tumorigenesis
Source: J Transl Med. 2023 Nov 17;21:826. doi: 10.1186/s12967-023-04611-8 (PMC10655258; doi:10.1186/s12967-023-04611-8)
Supplement: Supplementary file 2 — Additional file 2: Fig. S1. Expression of most palmitoylation-related genes is equal in various normal tissues. Fig. S2. Expression of palmitoylation-related genes is associated with cancer subtype and patient survival. Fig. S3. Mutation landscape of palmitoylation-related genes in human cancers. Fig. S4. CNV landscape of palmitoylation-related genes in human cancers. Fig. S5. Myc regulates palmitoylation. Fig. S6. Dysregulated palmitoylation is associated with immune infiltration in human cancers. Fig. S7. The expression levels of ZDHHC9 and ABHD17C genes are correlated with TMB and MSI. Fig. S8. Etoposide and piperlongumine are potential small molecules for regulating palmitoylation. Fig. S9. BI-2536, etoposide and piperlongumine regulate the expression of palmitoylation-related genes. Fig. S10. BI-2536, etoposide and piperlongumine are potential small molecules for targeting palmitoylation-related proteins. [file 12967_2023_4611_MOESM2_ESM.pdf]

Palmitoylation landscapes across human cancers reveal a role of palmitoylation in tumorigenesis

Yue Kong, Yugeng Liu, Xianzhe Li, Menglan Rao, Dawei Li, Xiaolan Ruan, Shanglin Li, Zhenyou Jiang, Qiang Zhang

Supplementary Figures

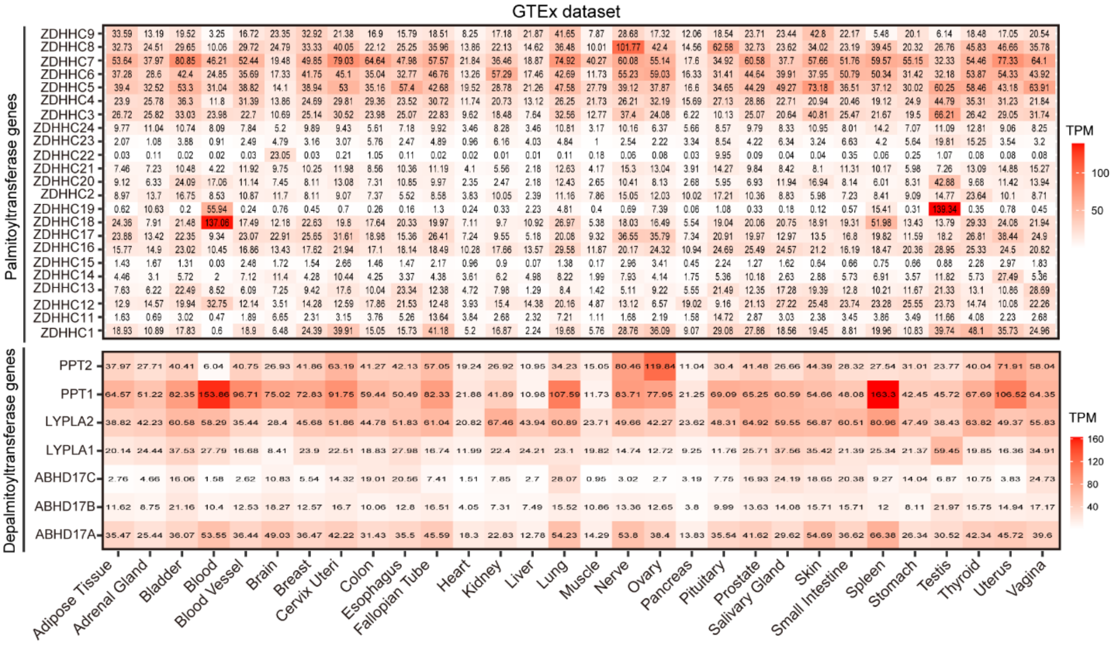

Fig. S1 Expression of most palmitoylation-related genes is equal in various normal tissues. Heatmap showing the expression of palmitoylation-related genes in different tissues from the GTEx database.

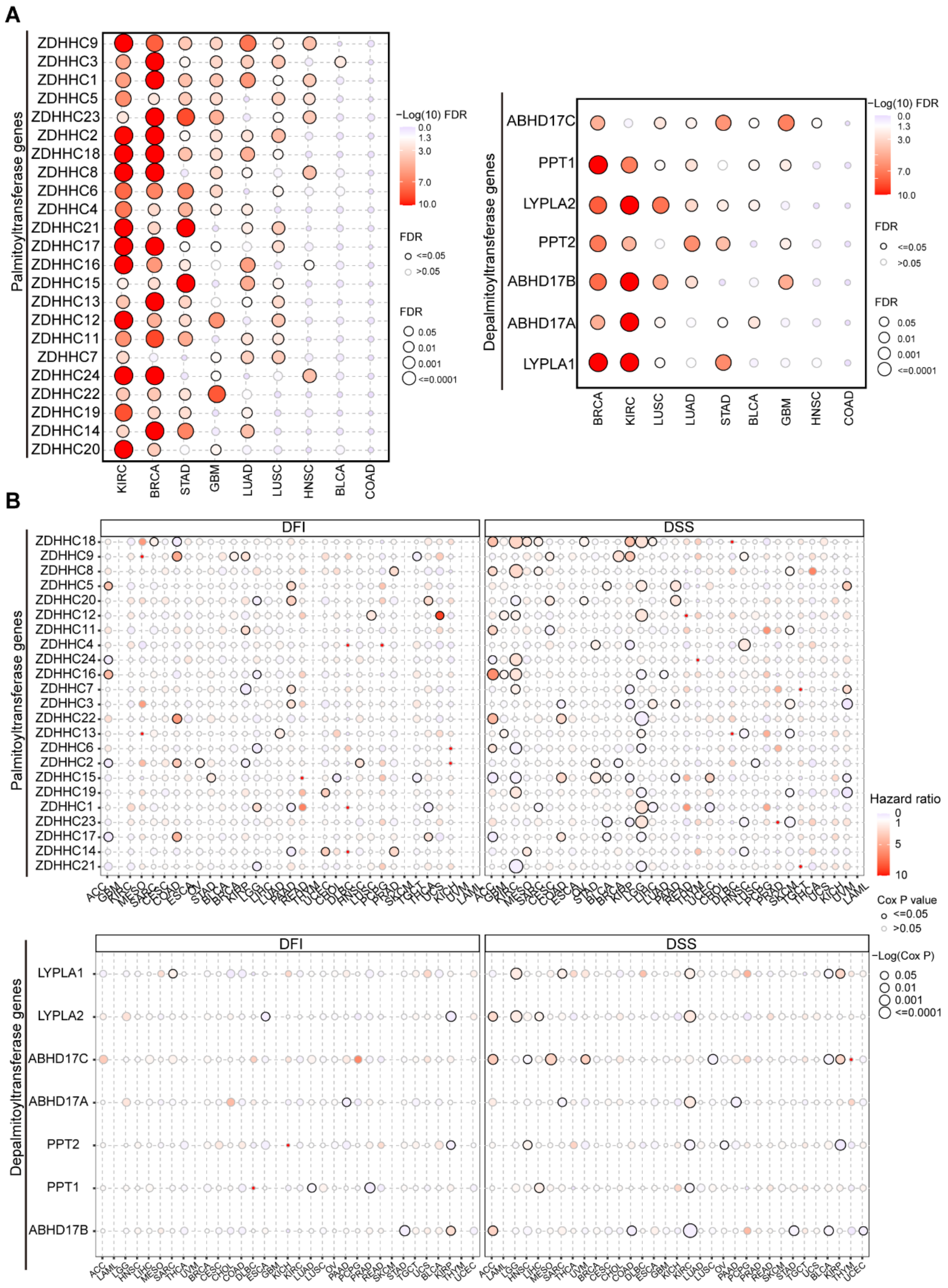

Fig. S2 Expression of palmitoylation-related genes is associated with cancer subtype and patient survival.

A Bubble plot showing the associations between cancer subtypes and expression of palmitoylation-related genes. Cancer subtype difference between high and low

expression of palmitoyl-acyltransferases (**Left**) or de-palmitoyl-acyltransferases (**Right**) genes.

B Bubble plot showing survival difference between the high and low expression of palmitoyl-acyltransferase (**Upper**) or de-palmitoyl-acyltransferase (**Lower**) genes.

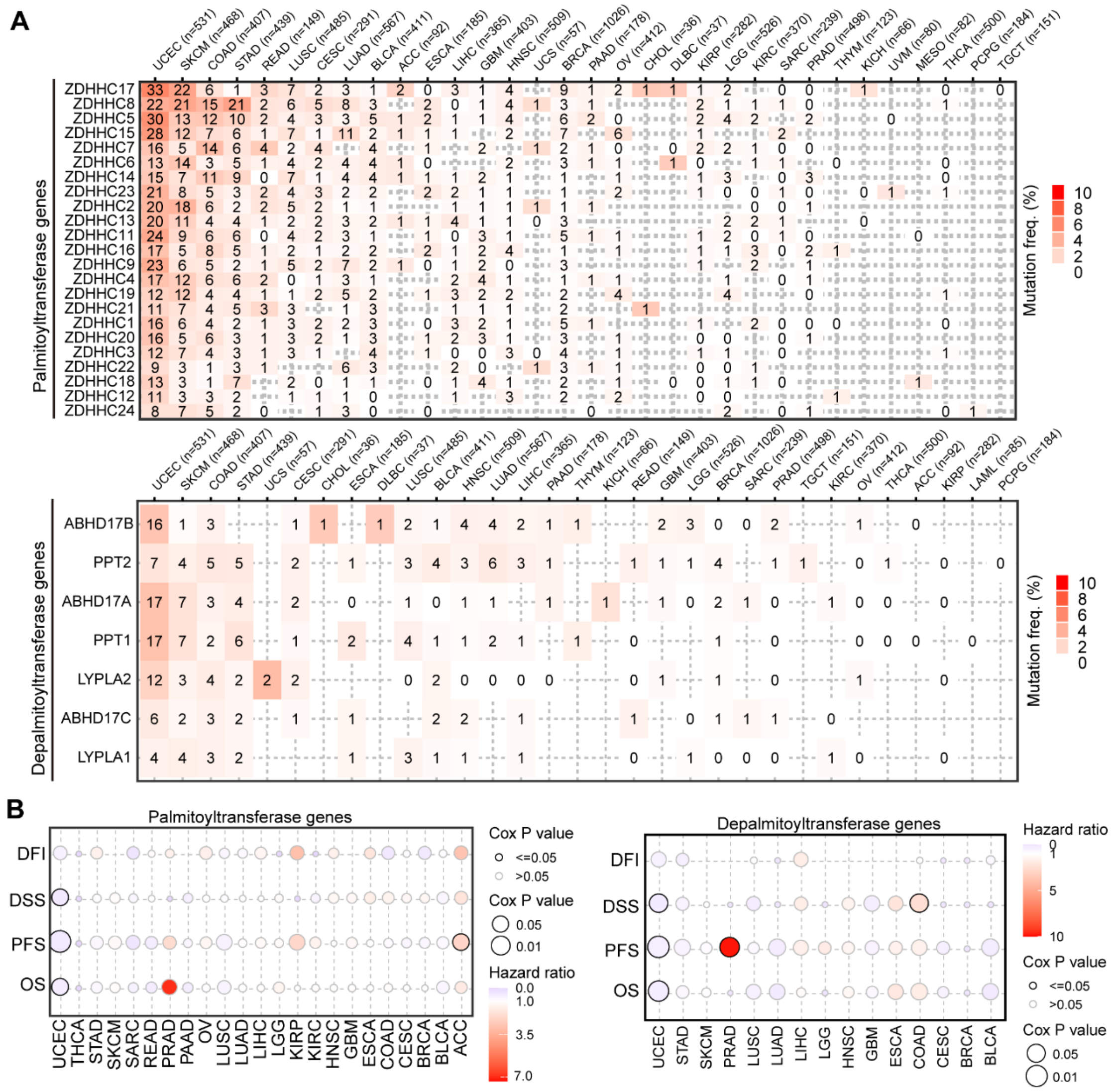

Fig. S3 Mutation landscape of palmitoylation-related genes in human cancers.

A Heatmap showing the mutation profile of palmitoyl-acyltransferases (**Upper**) or de-palmitoyl-acyltransferases genes (**Lower**) in human cancers from the TCGA database. **B** Bubble plot showing the survival difference between palmitoyl-acyltransferases (**Upper**) or de-palmitoyl-acyltransferases (**Lower**) gene set mutant and wide type in human cancers.

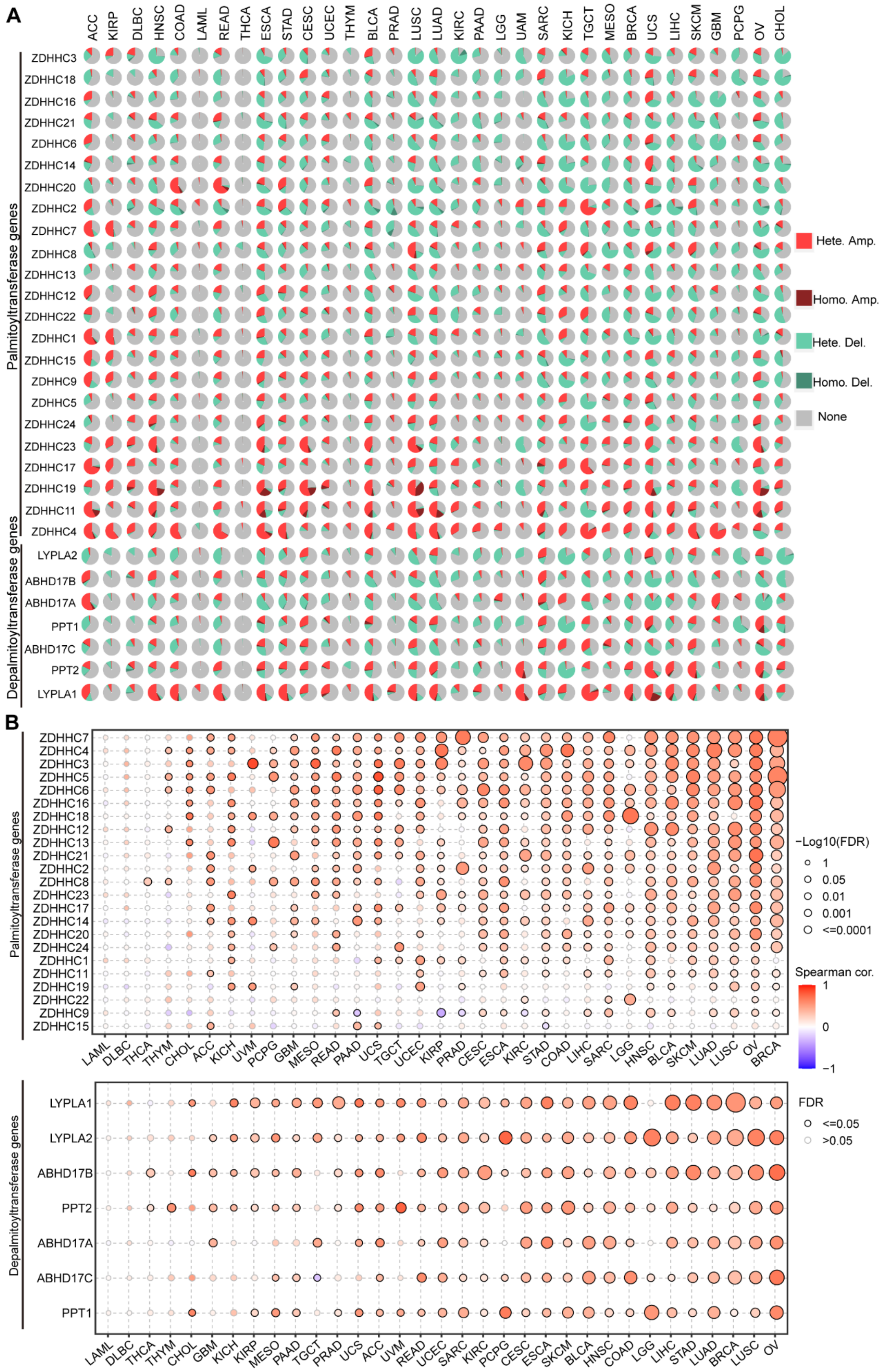

Fig. S4 CNV landscape of palmitoylation-related genes in human cancers.

A CNV percentage of palmitoyl-acyltransferase (**Upper**) or de-palmitoyl-acyltransferase (**Lower**) genes in human cancers.

B Correlations of CNV with mRNA expression of palmitoyl-acyltransferase (**Upper**) or de-palmitoyl-acyltransferase (**Lower**) genes in human cancers.

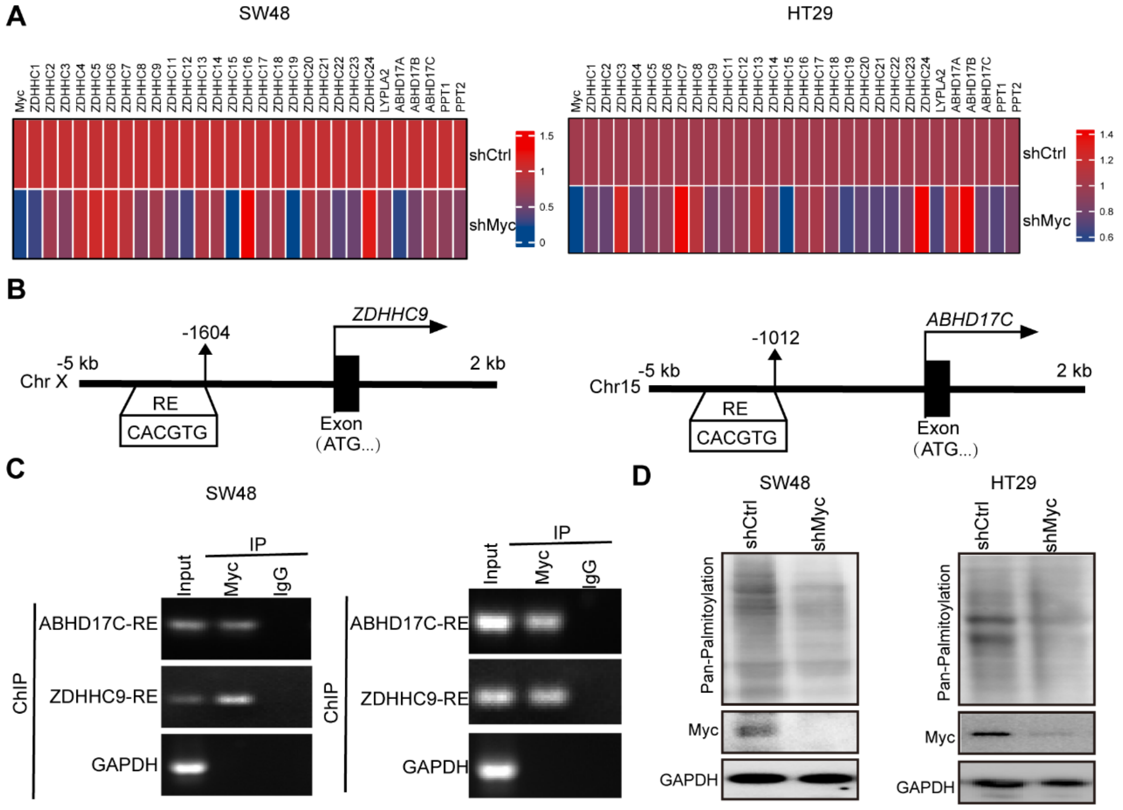

Fig. S5 Myc regulates palmitoylation.

A Heatmap showing the expression of palmitoyl-acyltransferases or de-palmitoyl-acyltransferases genes in Myc-depleted SW48 and HT29 cells.

B Diagram showing Myc occupancy on the promoters of ZDHHC9 and ABHD17C genes.

C Myc occupancy on the ZDHHC9 and ABHD17C promoters. ChIP was performed

using the endogenous Myc antibody in SW48 and HT29 cells. PCR analysis was conducted on the endogenous promoters of ZDHHC9 and ABHD17C genes.

D Global palmitoylation levels decrease upon Myc depletion in Myc-depleted SW48 and HT29 cells.

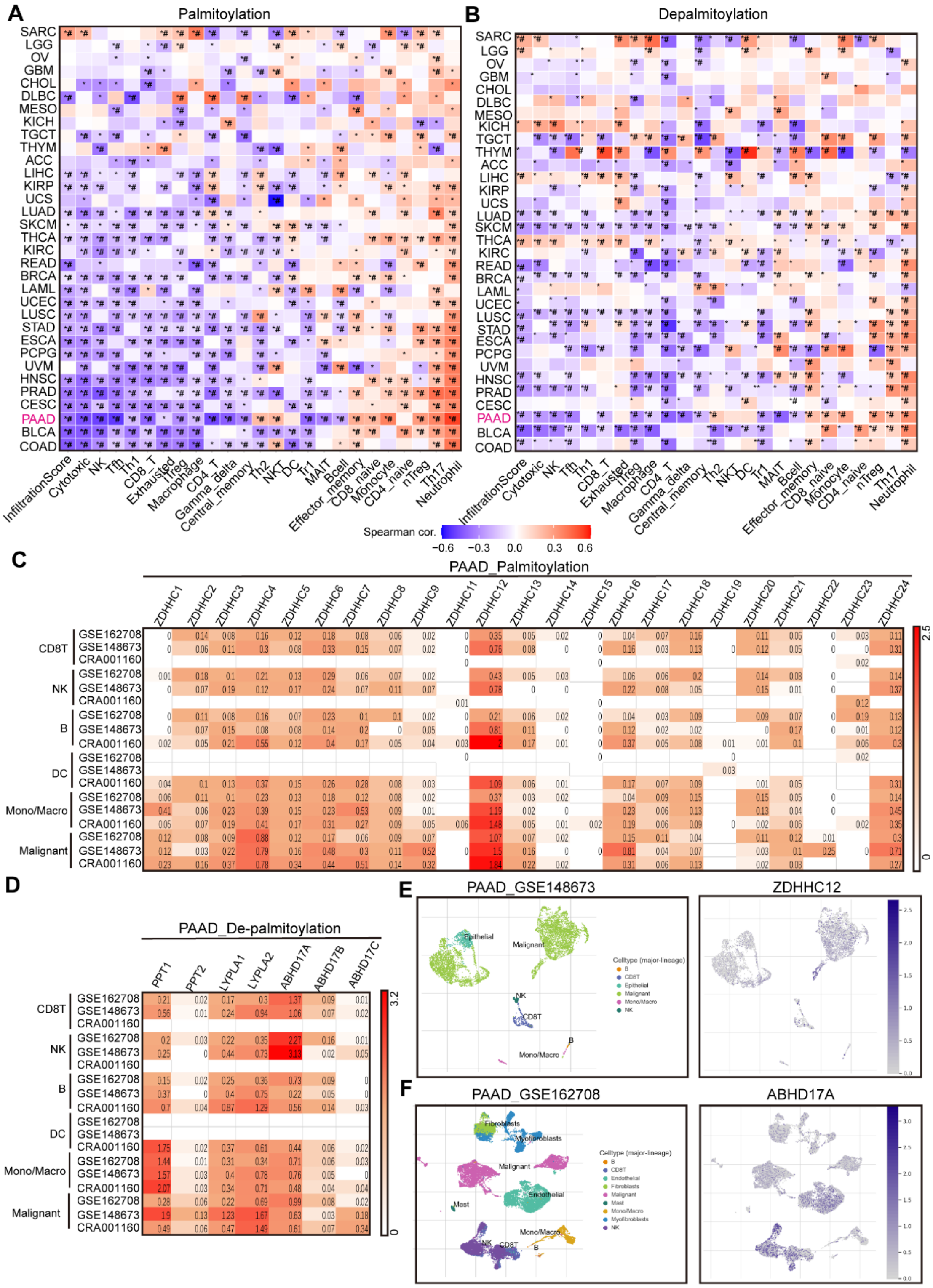

Fig. S6 Dysregulated palmitoylation is associated with immune infiltration in human cancers.

A, B Heatmap summarizes the significance of *P* value and *FDR* for the spearman correlation analysis between the GSVA score of palmitoyl-acyltransferases (**A**) or de-

palmitoyl-acyltransferases (**B**) gene set and immune cells' infiltrates.

C, **D** Heatmap of palmitoyl-Acyltransferases (**C**) or de-palmitoyl-Acyltransferases (**D**) gene expression in immune cells using single cell analysis.

E, **F** Analysis of ZDHHC12 (**E**) and ABHD17A (**F**) expression in immune cells of PPAD tumors using single cell datasets GSE148673 and GSE162708.

*P< 0.05; $^{\#}$FDR< 0.05.

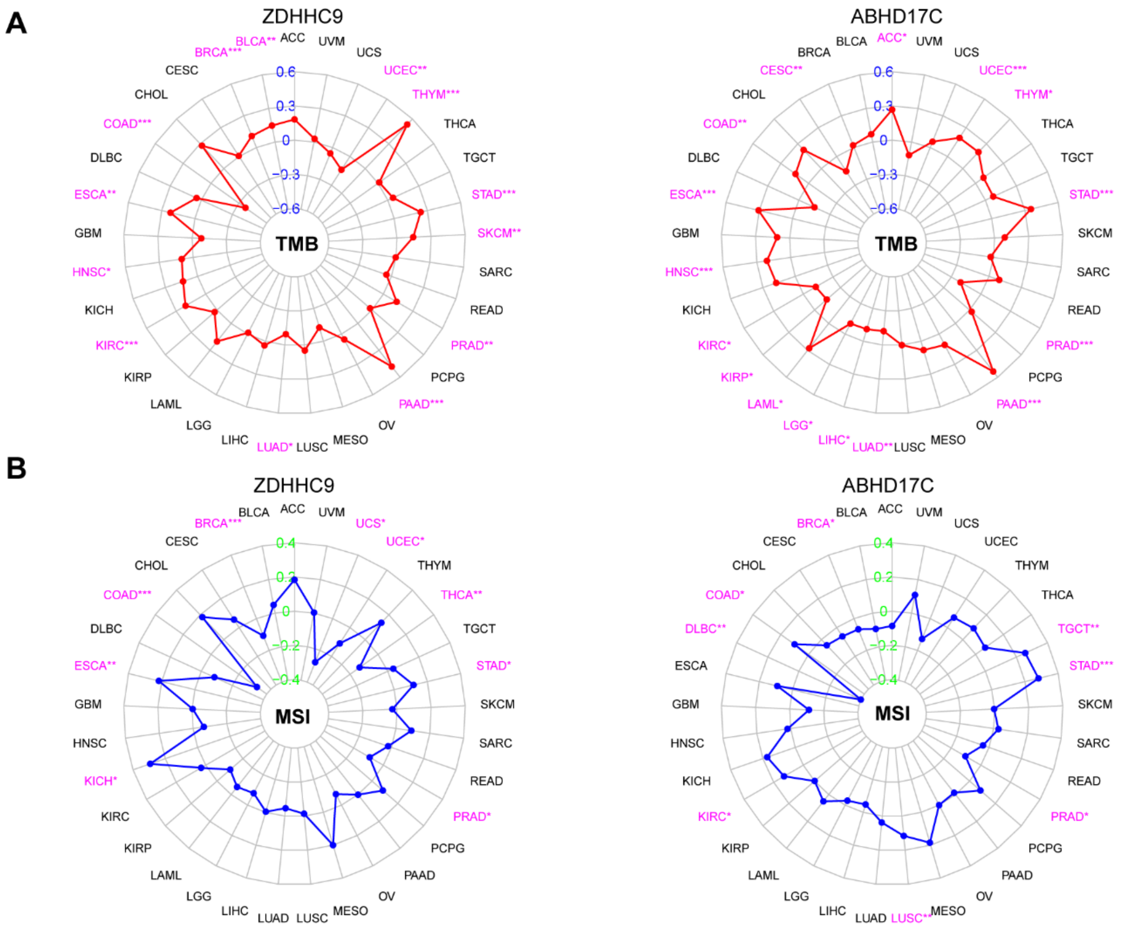

Fig. S7 The expression levels of ZDHHC9 and ABHD17C genes are correlated with TMB and MSI.

A, B Correlations between ZDHHC9 and ABHD17C gene expression and TMB (**A**) and MSI (**B**) in pan-cancer.

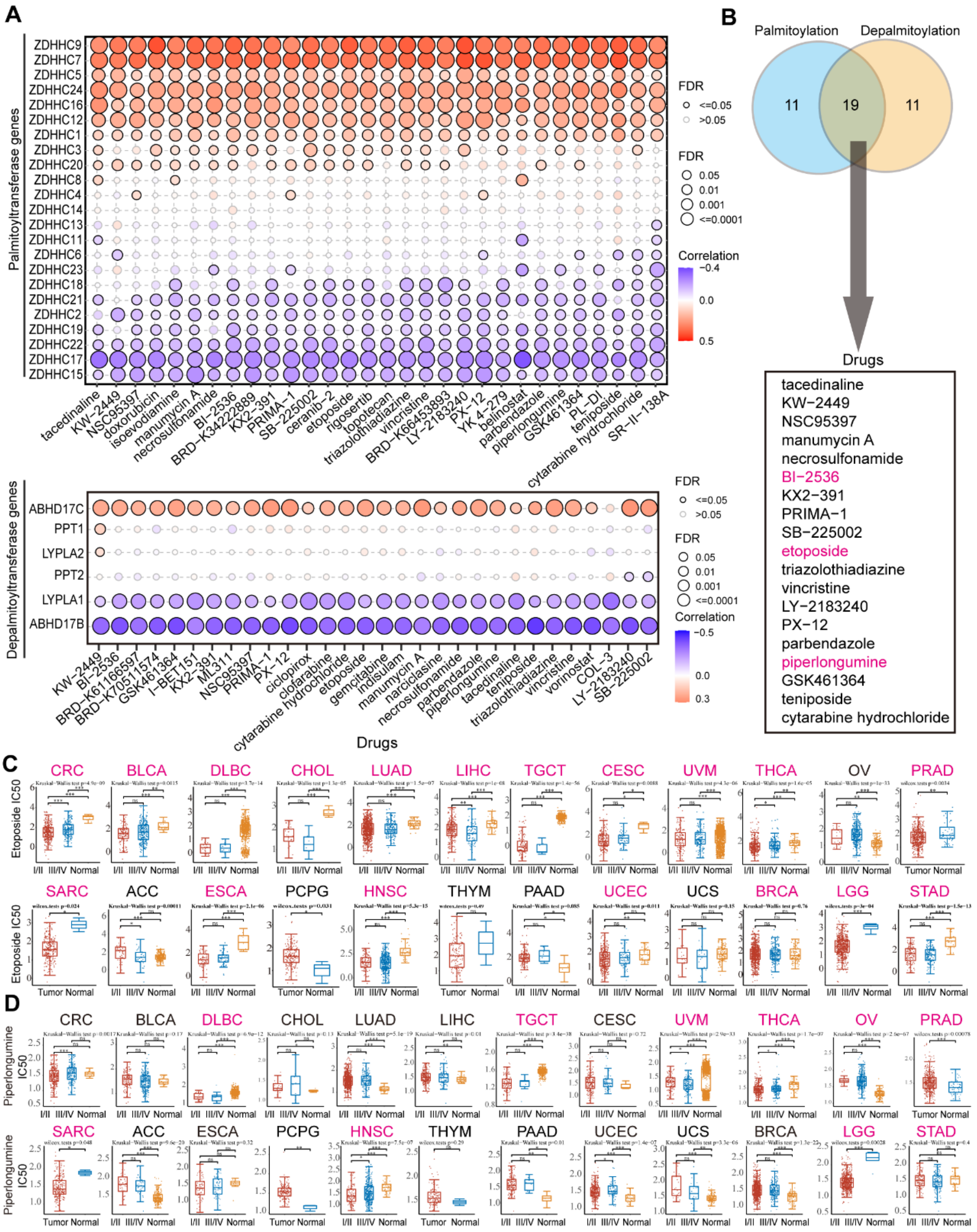

Fig. S8 Etoposide and piperlongumine are potential small molecules for regulating palmitoylation.

A Bubble plot showing the correlation between the expression of palmitoyl-acyltransferases (**Upper**) or de-palmitoyl-acyltransferases (**Lower**) genes and the sensitivity of CTRP drugs (top 30) in pan-cancer.

B Overlap analysis of potential small molecules regulating palmitoylation.

C, **D** Pan-cancer boxplot of etoposide (**C**) and piperlongumine (**D**) IC50 values in normal tissue and stage tumors.

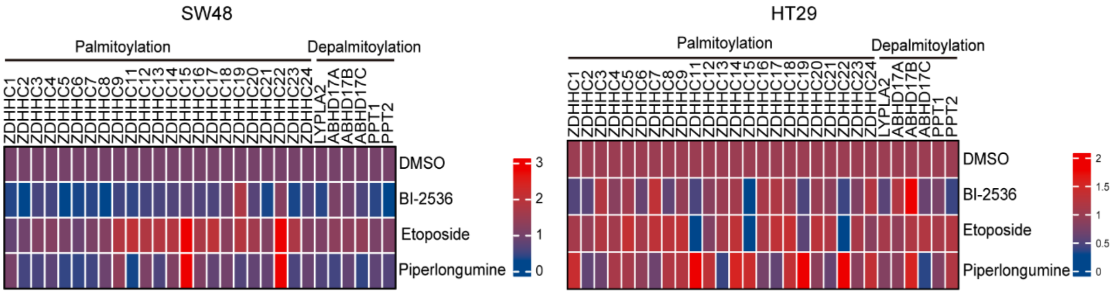

Fig. S9 BI-2536, etoposide and piperlongumine regulate the expression of palmitoylation-related genes.

Heatmap of palmitoyl-acyltransferases and de-palmitoyl-acyltransferases gene expression in BI-2536, etoposide, and piperlongumine-treated SW48 and HT29 cells.

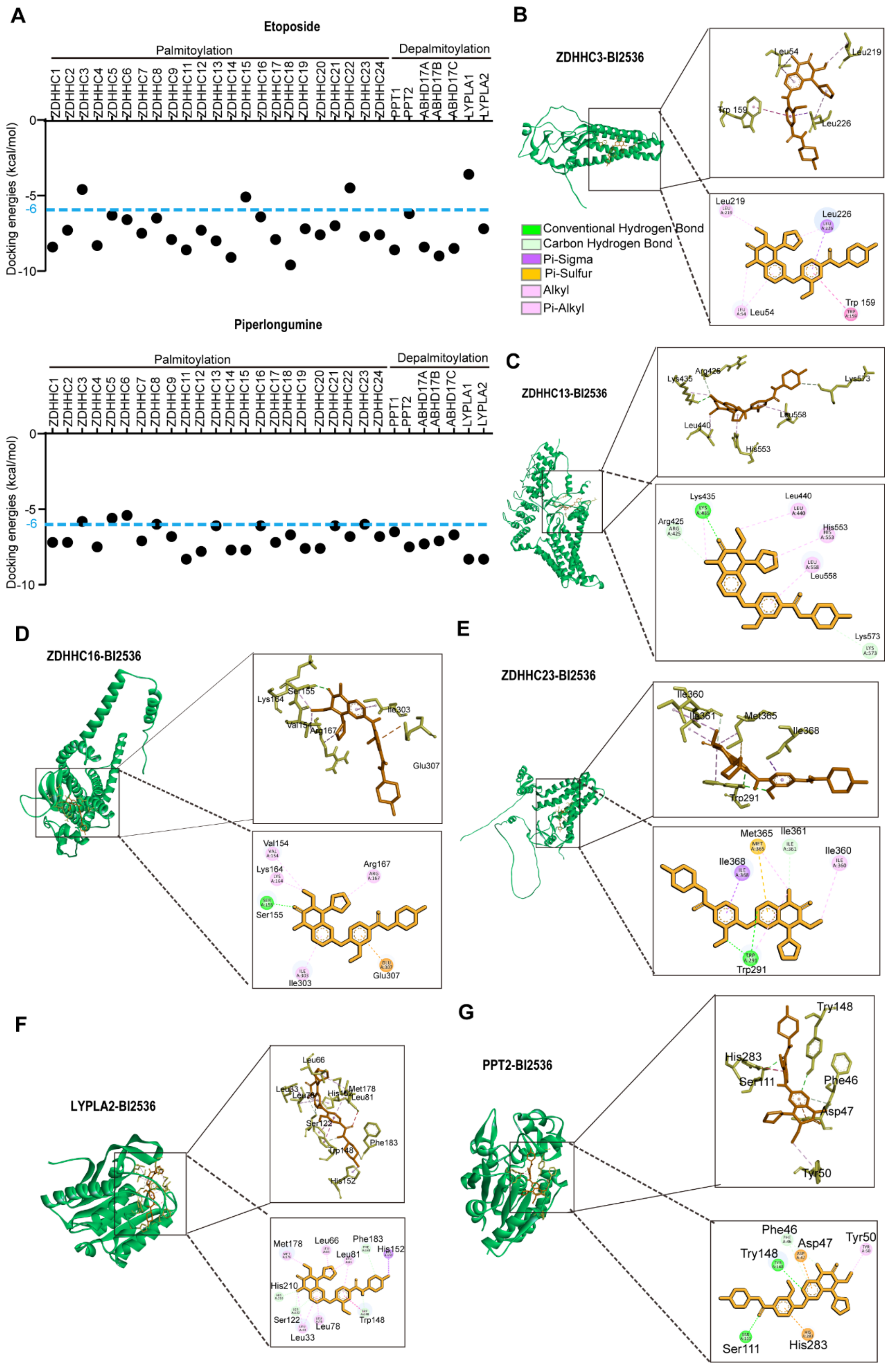

Fig. S10 BI-2536, etoposide and piperlongumine are potential small molecules for

targeting palmitoylation-related proteins.

A Scatter plot of docking energies between etoposide (**Upper**) and piperlongumine (**Lower**) and palmitoylation-related proteins.

B-G, Docking results of ZDHHC3 (**B**), ZDHHC13 (**C**), ZDHHC16 (**D**) ZDHHC23 (**E**), LYPLA2 (**F**) and PPT2 (**G**) proteins with small molecular compound BI-2536.
